# Theoretical Framework for the Study of Genetic Diseases Caused by Dominant Alleles

**DOI:** 10.3390/life13030733

**Published:** 2023-03-08

**Authors:** Michael F. Roberts, Stephen E. Bricher

**Affiliations:** 1 Department of Biology, Linfield University, McMinnville, OR 97128, USA; 2 Department of Mathematics and Computer Science, Linfield University, McMinnville, OR 97128, USA

**Keywords:** genetic disease, prevalence, penetrance, accurate diagnosis, disease iceberg effect, cumulative lifetime risk, familial aggregation

## Abstract

We propose a theoretical basis for analyzing several features of genetic diseases caused by dominant alleles, including: disease prevalence, genotype penetrance, and the relationship between causal genotype frequency and disease frequency. In addition, we provide a theoretical framework for accurate diagnosis and clinical approaches for disease study, including two examples in which inaccurate and incomplete diagnoses affect the estimates of disease prevalence: First, the disease iceberg effect shows that disease prevalence is often underestimated due to errors introduced by inaccurate diagnosis; second, because lifetime risk of disease is cumulative, and therefore an increasing function of age, measurements of prevalence are inaccurate if people of all ages are not included. Finally, we discuss the aggregation of genetic diseases. We identify theoretical and computational deficiencies associated with using the sibling recurrence-risk ratio as a measure of familial aggregation. We develop an alternative concept of aggregation and propose an associated measure that does not experience the deficiencies. Throughout, we provide clinicians and researchers practical implications of our theoretical framework.

## 1. Introduction

Determining the genetic basis for diseases is an important part of population genetics and epidemiology, as disorders can be caused both by a person’s genetic predisposition and by environmental influences. The accurate allocation of the cause between genes and the environment allows a better understanding of disease mechanisms and promotes techniques for diagnosing and combating disease [1,2].

The analysis of genetic diseases has a long history. Garrod [3] first drew attention to the relation between inheritance of recessive alleles and the appearance of alkaptonuria in human families. This work ultimately led to the understanding that body characteristics (phenotype) are primarily determined by cellular proteins and that genes (genotype) specify these proteins (e.g., enzymes). Genetic diseases are phenotypes; thus, a genetic disease is similar to any phenotype specified by a genotype [4]. Though different genetic diseases may have different biochemical bases, their transmission processes are identical, and each can be characterized as being caused by recessive or dominant alleles. We focus on single-gene disorders caused by dominant alleles and assume an autosomal “two-allele” model for the genotype-phenotype relationship; consequently, we will not discuss multi-gene or sex-linked diseases.

Our purpose is to clarify the relationship between disease-causing genotypes and the presence of the disease, as well as to clarify the role of accurate diagnosis. We identify theoretical and computational deficiencies associated with the current measure of familial aggregation and propose an alternative concept of aggregation and its measure. Our intention is therefore to describe the theoretical issues clearly, to show why accurate diagnosis is lacking in some cases, as well as to provide replacements for commonly used approaches that experience theoretical and computational deficiencies. Throughout, we provide clinicians and researchers practical implications of our theoretical framework.

In developing our theoretical framework, we will use: probability as a relative frequency; a set theoretic approach to probability; partitions and the law of total probability; conditional probabilities and their properties; population parameters and their estimators; and large-sample-size confidence intervals. As the background for the underlying probability and statistical concepts used, we recommend References [5,6].

Unbiased clinical studies can provide accurate estimates of population parameters (e.g., allele frequency, genotype penetrance, or disease prevalence), which are required for meaningful inferences about disease characteristics. Readers interested in specific protocols for obtaining unbiased clinical studies may see [7] for an in-depth discussion of clinical study design—including strategies for minimizing biases, the statistical analysis of the data, and ethical issues. In addition, we suggest two clinically oriented works that give additional perspectives on specific genetic diseases [1,2].

## 2. Disease-Causing Genotypes and Prevalence

We discuss disease-causing genotypes and their relationship to the presence of the associated disease caused by a dominant allele, including their role in determining the disease’s frequency in the population.

Traits (phenotypes) are divided into categories determined by genotypes written for convenience as if they consisted of only two alleles [8]. Indeed, most treatments of population genetics [9] focus on a two-allele model, while acknowledging a more complete treatment recognizes that genes have multiple alleles. Nonetheless, even genotype models describing more than two alleles [9] can be reduced to two-allele models if allele contribution is expressed in terms of the functions of the proteins synthesized by each allele.

Let *D* denote the event that an individual in the population has the disease caused by a dominant allele. Let P(D) denote the probability that any individual in the population has the disease. In the literature, P(D) is sometimes referred to as: (a) the *frequency* of the disease in the population; (b) the *risk* of the disease for an individual in the population; (c) the *likelihood* an individual in the population has the disease; or (d) the *prevalence* of the disease in the population [10,11].

In our two-allele model, we denote the alleles by *C* and *c* and define them as the only two options, where a *C* allele synthesizes a functioning protein and a *c* allele makes a non-functioning protein. The *C* allele is called a *dominant allele*, and the *c* allele is called a *recessive allele*. We will use the following notation for the frequency of these alleles in the population: Let p=P(C) denote the frequency (probability) of the *C* allele in the population; let q=P(c) denote the frequency (probability) of the *c* allele in the population. Obviously, p+q=1, since *C* and *c* are the only options in our two-allele model.

### 2.1. Penetrance and Environmental Influence

*Penetrance* refers to the frequency (probability) of the disease *D*, given a particular genotype CC, Cc, or cc [8,12,13,14]. Specifically, the penetrance of a particular genotype is the corresponding conditional probability: The penetrance of CC is P(D|CC); the penetrance of Cc is P(D|Cc), which is the same as the penetrance of cC; the penetrance of cc is P(D|cc). For example, P(D|CC) is the frequency of those in the population with genotype CC who have the disease *D*.

In agreement with some authors [15], we say a specific genotype has *full penetrance* provided its penetrance is one; for example, P(D|CC)=1 corresponds to the genotype CC having full penetrance. A specific genotype has *partial penetrance* provided its penetrance is less than one; for example, P(D|CC)<1 corresponds to the genotype CC having partial penetrance.

Penetrance is often presented in an imprecise manner [8], which may lead to misunderstanding; our probability-based quantitative description is unambiguous. Indeed, because genotype penetrance indicates the *frequency* of those people with a particular genotype who have the disease, penetrance is *not* a measure of disease severity. This means genotype penetrance does not influence whether a person has a severe, moderate, or mild form of the disease. For example, P(D|Cc)=0.5 means that, of those people with genotype Cc, about 50% are identified with the disease; it does *not* mean a diseased person with genotype Cc has a moderate form of the disease. Disease severity is instead related to the concepts of “complete/incomplete dominance” and “expressivity” [8].

The concept of penetrance is one way to include an environmental component in the genotype-phenotype correlation. The estimate of penetrance may include a suspected environmental effect on gene expression (e.g., eating gluten is necessary for the onset of Celiac disease [16]). Even so, it is not always possible to accurately identify a disease phenotype, though the genotype might be known. Griffiths et al. [8] describe this as an aspect of penetrance leading to the “subtlety” of the mutant phenotype; we add that incomplete diagnosis can masquerade as partial penetrance (Section 3).

In order to use genotype frequencies to accurately estimate disease prevalence, it is *essential* that penetrance be accurately estimated (Section 2.2). With that in mind, it is important to note that using clinical studies to estimate the penetrance of a particular genotype requires: (i) the use of a genetic test to identify whether a person has the genotype; (ii) the identification of the disease’s phenotypes; and (iii) the use of a diagnostic test to determine whether such a person with the genotype has the disease (i.e., exhibits the disease’s phenotypes). Thus, the accuracy of diagnosis plays a critical role in estimations of penetrance (Section 3).

### 2.2. Prevalence of Diseases Caused by Dominant Alleles

A person with either genotype CC or Cc might be affected with a condition sometimes called a *dominant disorder* [15]. This may occur where the genotype cc produces the wild-type phenotype, but mutation from *c* to *C* generates a new version of the *c* protein that may impair cellular function.

We introduce a parameter that describes the relationship between the penetrance of CC and Cc. The parameter *r* is the ratio of the penetrance of Cc to the penetrance of CC (Section 2.1); that is,

(1) $$r=\frac{P(D|Cc)}{P(D|CC)}$$

where 0<r≤1 because 0<P(D|Cc)≤P(D|CC). We will use the parameter *r* in clarifying a theoretical framework for the prevalence of diseases caused by dominant alleles.

Because the genotypes CC, Cc, cC, and cc form a partition of the population, prevalence can be written in the form

(2) $$P\left(D\right)=p^{2}P\left(D|CC\right)+2pqP\left(D|Cc\right)+q^{2}P\left(D|cc\right).$$

Equation (2) describes the prevalence for diseases (P(D)) in terms of the frequencies of the alleles (*p* and *q*) and in terms of the penetrance of the genotypes (P(D|CC), P(D|Cc), and P(D|cc)). The derivation of Equation (2) is provided in Appendix A.

For a disease caused by a dominant allele, P(D|cc)=0, P(D|CC)>0, and P(D|Cc)>0. In this case, Equation (2) becomes

$$P\left(D\right)=p^{2}P\left(D|CC\right)+2pqP\left(D|Cc\right);$$

in other words, disease prevalence (P(D)) in principle equals the sum of the homozygote dominant and heterozygote genotype population frequencies ($p^{2}$ and 2pq), where each frequency is *rescaled* according to its associated penetrance (P(D|CC) and P(D|Cc)). This allows us to introduce a new formulation for P(D). Substituting Equation (1) yields,

$$\begin{array}{cc} P(D) & =p^{2}P\left(D|CC\right)+2pqrP\left(D|CC\right)\\ \; & =\left(p^{2}+2pqr\right)P\left(D|CC\right)\\ \; & =p(p+2qr)P(D|CC)\\ \; & =p(p+2(1-p)r)P(D|CC)\\ \; & =p(p+2r-2pr)P(D|CC), \end{array}$$

which we write in the form

(3) P(D)=p(2r+(1−2r)p)P(D|CC).

Incidentally, in the above derivation of Equation (3), we demonstrate that

$$p^{2}+2pqr=p\left(2r+\left(1-2r\right)p\right);$$

in other words, the expression p(2r+(1−2r)p) is simply another way to write the sum of the homozygote dominant and (rescaled by *r*) heterozygote population frequencies ($p^{2}$ and 2pqr). The advantages of using this expression will become apparent in the following discussion.

Equation (3) completely characterizes the theoretical prevalence of such a disease by describing it in terms of only three parameters: the penetrance of the genotype CC (P(D|CC)); the parameter *r* (Equation (1)); and the frequency of the *C* allele in the population (p=P(C)). It thus identifies the roles of the three important parameters in determining disease prevalence. In particular, prevalence has a different structure as a function of *p* in each of the three cases for *r*:

(i) If 1/2<r≤1, then P(D) has a concave down parabolic relationship in terms of *p*.
(ii) If r=1/2, then P(D) has a linear relationship in terms of *p*.
(iii) If 0<r<1/2, then P(D) has a concave up parabolic relationship in terms of *p*.

**Figure 1:**
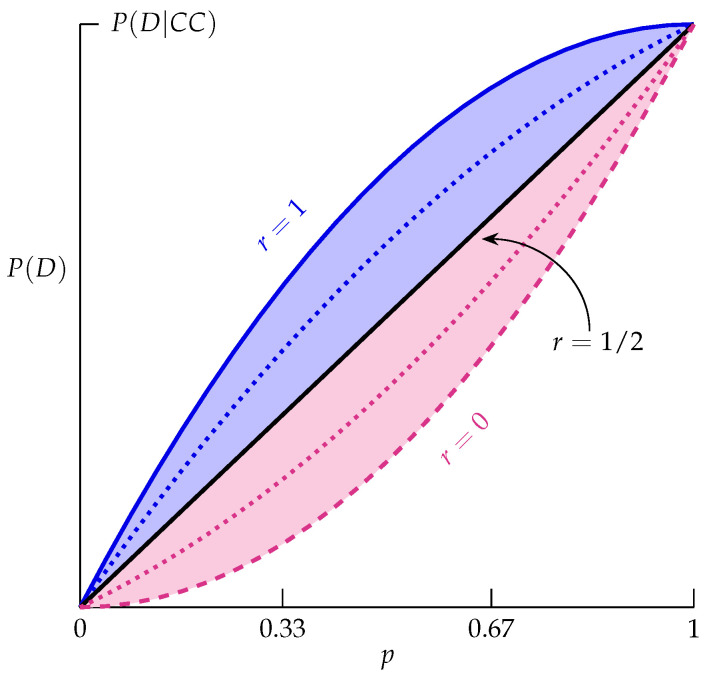
Graph of disease prevalence P(D) versus dominant allele population frequency *p* for the three cases of *r* (Equation (3)): (i) the blue shaded region corresponds to 1/2<r≤1, where the solid blue curve is r=1, and the dotted blue curve is an illustrative example (r=3/4); (ii) the black line corresponds to r=1/2; (iii) the red shaded region corresponds to 0<r<1/2, where the dashed red curve is the lower limit r=0, which cannot be achieved because r>0 for dominant diseases. The dotted red curve is another illustrative example (r=1/4). The theoretical prevalence of *any* disease caused by a dominant allele must be above the dashed red curve and, at most, the solid blue curve. Numerical values on the vertical axis can be assigned once a value of P(D|CC) is known. Note that the largest possible value of P(D) is P(D|CC), which occurs at p=1, where all three cases coalesce.

Figure 1 illustrates how Equation (3) uses allele frequency and the penetrance of the genotype CC to determine disease prevalence, where graphs for the three cases of *r* are shown:

(i) The blue shaded region corresponds to 1/2<r≤1, where the solid blue curve is r=1, and the dotted blue curve is an illustrative example (r=3/4).
(ii) The black line corresponds to r=1/2.
(iii) The red shaded region corresponds to 0<r<1/2, where the dashed red curve is the lower limit r=0, which cannot be achieved because *r* must be positive for diseases caused by dominant alleles. The dotted red curve is another illustrative example (r=1/4).

An advantage of Equation (3) (and Figure 1) over other expressions for prevalence (e.g., Equation (2)) is that it clearly identifies the critical role *r* plays in determining the prevalence’s different theoretical framework as a function of *p* in each of the three cases mentioned. Incidentally, the parameter *r* has an important role in our alternative new concept of disease aggregation (Section 4).

An important property for the prevalence of diseases caused by dominant alleles illustrated in Figure 1 is:

The theoretical prevalence of *any* disease caused by a dominant allele must be greater than the dashed red curve (r=0) and, at most, the solid blue curve (r=1). That is, P(D) always satisfies

$$p^{2}P\left(D|CC\right)<P\left(D\right)\leq p\left(2-p\right)P\left(D|CC\right).$$

Thus, if clinicians estimate a value of disease prevalence ($\hat{P}\left(D\right)$) to be outside this interval, it should suggest to them that there likely are diagnostic errors (Section 3) with how P(D) has been estimated.

Moreover, if a disease is thought to be caused by a dominant allele, then clinicians should find that prevalence estimated from diagnostic tests will be close to P(D) described in Equation (3). If it is not, then that should alert clinicians that the diagnostic test is possibly not accurate (Section 3.2).

### 2.3. Necessary and/or Sufficient Genotypes

We develop the theoretical framework characterizing when the disease-causing genotypes are necessary and/or sufficient for the presence of the disease. Let *G* denote the disease-causing genotypes for a disease caused by a dominant allele; specifically, G={CC}∪{Cc}.

To define the logical concepts of “necessary” and “sufficient”, we frame the discussion in terms of the events *G* and *D* representing the disease-causing genotypes and the presence of the disease, respectively. However, the concepts apply to any two events; for example, in Section 3.2, we discuss whether a positive result in a diagnostic test (denoted by *T*) is necessary and/or sufficient for the presence of the disease (again, denoted by *D*).

We say that *G* is *necessary* for *D* provided D⇒G. That is, the occurrence of *D* implies the occurrence of *G*. In other words, (in this context) if a person has the disease, then the person will (likely) have the disease-causing genotype.

We say that *G* is *sufficient* for *D* provided G⇒D. That is, the occurrence of *G* implies the occurrence of *D*. In other words, (in this context) if a person has the disease-causing genotype, then the person will (likely) have the disease.

**Conditional probability formulations**. We now develop equivalent conditional probability formulations for the concepts of “necessary” and “sufficient” discussed above. The formulations apply to any two events, but we will frame the discussion in terms of *G* and *D* as above (see Section 3.2 for another example). Observe that P(G|D)=1 is equivalent to saying that “*G* is *necessary* for *D*”. Also, observe that P(D|G)=1 is equivalent to saying that “*G* is *sufficient* for *D*”. The details for the equivalence of these formulations is established in Appendix B.

We now use the formulations to clearly identify when the disease-causing genotypes are necessary and/or sufficient for the presence of a disease caused by a dominant allele. In addition, we include implications for clinicians as the context.

For a disease caused by a dominant allele, $P(D|G^{\prime })=0$. Now,

$$\begin{array}{cc} P(D) & =P\left(D\cap G\right)+P\left(D\cap G^{\prime }\right)\\ \; & =P\left(D\cap G\right)+P\left(D|G^{\prime }\right)P\left(G^{\prime }\right)\\ \; & =P(D\cap G), \end{array}$$

which implies

$$P\left(G|D\right)=\frac{P(D\cap G)}{P(D)}=\frac{P(D)}{P(D)}=1;$$

therefore, *G* is necessary for *D*. Moreover,

$$P\left(D|G\right)=\frac{P(D\cap G)}{P(G)}=\frac{P(D)}{P(G)}$$

hence, *G* is sufficient for *D* if and only if P(D)=P(G). Recall that the frequency of the disease-causing genotypes is

$$P\left(G\right)=P\left(CC\right)+P\left(Cc\cup c\;C\right)=p^{2}+2pq\;;$$

therefore, by Equation (2) (since 0<P(D|CC)≤1 and 0<P(D|Cc)≤1), we conclude

P(D)=P(G)⇔P(D|CC)=1andP(D|Cc)=1.

Thus, P(D|G)=1 if and only if P(D|CC)=1 and P(D|Cc)=1.

In summary, the disease-causing genotypes CC and Cc are *always* necessary for *D*; they are sufficient for *D if and only if* the disease-causing genotypes are fully penetrant (P(D|CC)=1 and P(D|Cc)=1).

An implication for clinicians is that if they believe the disease-causing genotypes are “necessary, but not sufficient” for the presence of the disease, then P(D|CC)≠1 and/or P(D|Cc)≠1. Two explanations are: there could be other components (e.g., environmental) affecting the presence of the disease, resulting in CC and/or Cc not being fully penetrant; or it could be that the associated diagnostic test lacks the accuracy (Section 3.2) to correctly predict that the genotypes are fully penetrant. Consequently, it is essential that clinicians not use their belief that a disease-causing genotype is partially penetrant as the justification for relying on an inaccurate diagnostic test. In all of these scenarios, it is imperative that clinicians continue their investigations, ultimately seeking a thorough understanding and explanation of the actual relationship between P(D) and P(G).

In Section 3, we provide a similar analysis with *D* and a diagnostic test’s positive result, which we denote by *T*. Specifically, we demonstrate that accurate diagnosis is equivalent to *T* being necessary and sufficient for *D*. This allows us to develop, in Section 2 and Section 3, a unified theoretical framework for identifying a genetic disease caused by a dominant allele, as summarized in Section 5.

## 3. The Role of Diagnostic Tests

We provide three fundamental concepts for obtaining accurate estimates of disease prevalence: (1) identifying the genetic basis for the disease (Section 3.1); (2) achieving an accurate diagnosis via appropriate tests (Section 3.2); and (3) viewing disease prevalence as a cumulative lifetime risk [11] (Section 3.3).

**Figure 2:**
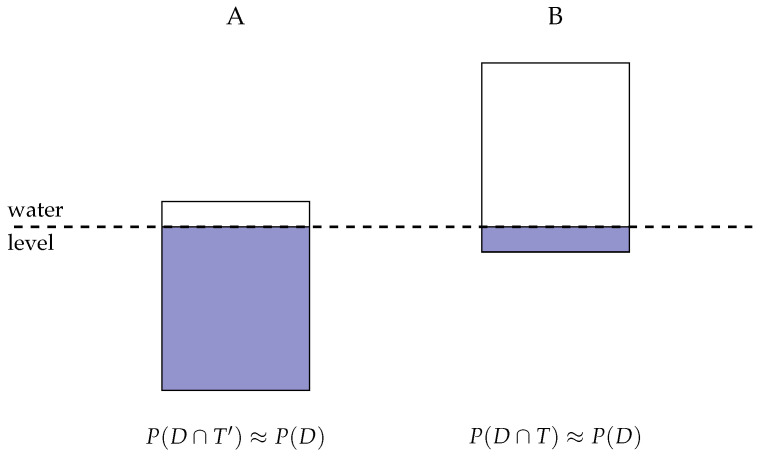
An extended disease iceberg analogy differentiating between various levels of identifying a disease based on a particular diagnostic test. Each rectangle (an iceberg) represents the proportion of the population with a given disease (P(D)) and is the same in each panel. The differences between the panels represent the various abilities that particular diagnostic tests may have in identifying the disease. The white region (above water portion) in each rectangle denotes the proportion of the population with the disease and a positive test result (P(D∩T)), while the blue region (below water portion) in each rectangle denotes the proportion of the population with the disease, but unknown because they have a negative test result ($P(D\cap T^{\prime })$). (**A**) A classical disease iceberg effect in which most of those with the disease are undiagnosed. (**B**) A well-identified disease in which almost all of the proportion of the population with the disease has a positive test result.

Before discussing the three fundamental concepts, it is important to recognize that the prevalence of genetic diseases is commonly underestimated [17,18,19,20,21]. This general under-diagnosis of diseases occurs because of inattention to the three fundamental concepts, and specifically because of the difficulty of identifying people with genetic diseases that are either non-lethal or that have symptoms similar to those of other diseases. Last [17] conceived of the analogy of a *disease iceberg* to describe this general disparity between the perceived and actual prevalence of a disease in the population. In his model, the entire iceberg represents the proportion of the population with the disease (actual prevalence); the “above water portion” of the iceberg corresponds to the diagnosed portion of the population with the disease (perceived prevalence); the “below water portion” of the iceberg corresponds to the portion of the population with the disease, but as yet undiagnosed (Figure 2A).

**Theoretical framework**. Let *D* denote the event that an individual from the population has the disease. Let *A* denote the event that an individual from the population has been diagnosed with the disease. The complement of *A* (denoted by $A^{\prime }$) will therefore be the event that an individual from the population has not been diagnosed with the disease for whatever reason.

Consider a disease with a *significant iceberg effect*, that is to say, the above-water portion of the iceberg is *significantly smaller than* the below-water portion of the iceberg. For the diseases studied by Last [17], the undiagnosed cases were 2–10-times the diagnosed cases. In other words,

$$P\left(D\cap A\right)\ll P\left(D\cap A^{\prime }\right).$$

Now, $D=\left(D\cap A\right)\cup \left(D\cap A^{\prime }\right)$, and because *A* and $A^{\prime }$ are mutually exclusive, $P\left(D\right)=P\left(D\cap A\right)+P\left(D\cap A^{\prime }\right)$. Hence, for a disease experiencing a significant iceberg effect,

P(D∩A)≪P(D),

demonstrating that the perceived prevalence (P(D∩A)) consisting of those thought to be affected by the disease will significantly underestimate the actual disease prevalence (P(D)).

The disease iceberg effect is common among diseases caused by dominant alleles and can be significant; indeed, disease prevalence can be underestimated by close to 90% [17,19,22]. Moreover, knowing the ratio of diagnosed-to-undiagnosed cases allows researchers and clinicians to more accurately estimate the actual disease prevalence P(D) [17,19,22], which we illustrate with an example.

Consider a disease with a perceived prevalence of 3.6% (P(D∩A)=0.036). In addition, suppose it is reported that 90% of those with the disease are undiagnosed; that is, $P\left(D\cap A^{\prime }\right)=0.9P\left(D\right)$. Using this information, researchers and clinicians can give a more accurate estimate of the actual disease prevalence P(D). Indeed, one can show that P(D)=0.36; thus, the actual disease prevalence is more accurately estimated as 36%, which is 10-times the perceived prevalence.

In Section 3.1, we extend the iceberg analogy and explain that the disease iceberg effect can be reduced by better: (i) disease identification; (ii) knowledge of disease-causing genotypes; and (iii) diagnosis (Section 3.2).

### 3.1. Identifying a Genetic Disease

Identifying a genetic disease requires two key approaches: (i) the assignment of a disease to a particular genotype; and (ii) the performance of accurate diagnostic tests.

**The assignment of a disease to a particular genotype**. Each person with the disease caused by a dominant allele has a particular genotype (CC or Cc). This genotype can be inferred from a family pedigree, and it can be directly determined by laboratory genotype tests. The genotype can be correlated via other laboratory tests with known symptoms and signs of the disease in order to discover (structurally, immunologically, or physiologically) why the particular genotype generates the disease phenotype. A combination of genetic tests and diagnostic tests is used; these tests must each be sensitive (very high true-positive rate) and specific (very high true-negative rate) for an accurate assignment (Section 3.2). If the various tests are appropriate and accurate, they should all agree with each other within reasonable error bounds. If different tests give different results regarding disease presence, clinicians should determine why the tests differ. These tests plus careful clinical examination should lead to an accurate diagnosis that minimizes the likelihood of misidentification.

**The performance of accurate diagnostic tests**. Clinical studies are used to estimate disease prevalence (Section 2.2), to determine which symptoms and signs are the most relevant, and to correlate these with the genotypes of disease carriers. Medical diagnoses (e.g., physical biopsies, tests for antibodies, and observation of symptoms) are combined with genotype determination [23].

**Theoretical framework**. Let *T* denote the event that a particular diagnostic test yields a positive result for the disease, which can be used to decompose P(D) as

$$P\left(D\right)=P\left(D\cap T\right)+P\left(D\cap T^{\prime }\right).$$

We develop an *extended disease iceberg analogy* to differentiate between various levels of identifying a disease based on a particular diagnostic test. In Figure 2, the rectangle (an iceberg) in each panel represents the proportion of the population with a given disease. In our analysis, both the disease and P(D) are the same in both panels. The differences between the panels represent the various abilities that particular diagnostic tests may have in identifying the disease. The white region (above water portion) in each rectangle denotes the proportion of the population with the disease and a positive test result (P(D∩T)), while the blue region (below water portion) in each rectangle denotes the proportion of the population with the disease, but who are unknown because they have a negative test result ($P(D\cap T^{\prime })$).

More precisely, we have the following levels of a diagnostic test identifying a disease:

- A disease is *not a well-identified disease* (with respect to the diagnostic test) provided
  $$P\left(D\cap T^{\prime }\right)\approx P\left(D\right),$$
  which is equivalent to P(D∩T)≈0. This implies the prevalence of the disease will be significantly underestimated by the diagnostic test and is equivalent to Last’s [17] concept of the *disease iceberg effect* (Figure 2A).
- A disease is *a well-identified disease* (with respect to the diagnostic test) provided
  P(D∩T)≈P(D),
  which is equivalent to $P(D\cap T^{\prime })\approx 0$. This implies the diagnostic test will yield an accurate estimator, via an unbiased clinical study based on the diagnostic test, for the prevalence of the disease (Figure 2B).

The clinical understanding of diseases has progressed over time based on improvements in the understanding of disease mechanisms and also on the development of new diagnostic tools. Thus, we suggest that the panels for the hypothetical disease in Figure 2 should illustrate the progression from “not well-identified” to “well-identified” in an actual disease as diagnostic tests improve in disease identification. In Section 3.2 and Section 3.3, we develop a theoretical framework for achieving this, as well as include suggestions/implications for researchers and clinicians.

Dominant fatal diseases, such as Huntington’s disease, have a clear genotype-phenotype relationship and straight-forward diagnostic approaches; they should, therefore, show minimal iceberg effects—they are “well-identified” diseases (Figure 2B). For others, such as prion diseases [13], the genotype-phenotype relation is not as well identified (Figure 2A). Prion diseases are rare disorders in which abnormally folded proteins cause neural disabilities. An example is Creutzfeldt-Jacob disease [24], in which the disease-causing protein originates from an alteration in allele sequence or is obtained from an exogenous source (e.g., the diet). Only the genetic version of the disorder is relevant here.

### 3.2. Accurate Diagnosis

Again, we let *D* be the event that an individual in the population has the disease and let *T* be the event that a diagnostic test yields a positive result for the disease. For example, a diagnostic test might be: (i) a biopsy; (ii) a test for blood-borne substances, such as antibodies associated with the disease; or (iii) a test based on the presence of symptoms associated with the disease [2].

Recall that *D* and *T* can be used to partition a group of individuals (e.g., the population as a whole or a clinical study corresponding to a random sample of a population under consideration) of size *n* as shown in Table 1, where:

$$\begin{array}{cc} n_{11} & =\mathrm{the}\;\mathrm{number}\;\mathrm{with}\;D\;\mathrm{and}\;T;\\ n_{12} & =\mathrm{the}\;\mathrm{number}\;\mathrm{with}\;D\;\mathrm{and}\;T^{\prime };\\ n_{21} & =\mathrm{the}\;\mathrm{number}\;\mathrm{with}\;D^{\prime }\;\mathrm{and}\;T;\\ n_{22} & =\mathrm{the}\;\mathrm{number}\;\mathrm{with}\;D^{\prime }\;\mathrm{and}\;T^{\prime }; \end{array}$$

and $n=n_{11}+n_{12}+n_{21}+n_{22}$.

In addition, recall that *the accuracy of the diagnostic test* is defined to be

$$\mathrm{Accuracy}=\frac{n_{11}+n_{22}}{n},$$

which measures the frequency of those individuals in the clinical study that are correctly diagnosed. The closer the ratio is to one, the more accurate the diagnostic test is. Only a diagnostic test with $n_{12}\approx 0$ and $n_{21}\approx 0$ will provide an accurate diagnosis (Accuracy≈1). We now discuss the properties of such a test.

#### 3.2.1. Necessary and Sufficient Diagnostic Tests

We show that accurate diagnosis is equivalent to a positive test result being *both* necessary and sufficient for the presence of the disease. Establishing this equivalence leads to several new advances: (i) we will be able to describe the theoretical mechanism for developing an accurate diagnosis (Section 3.2.2); (ii) we will be able to develop a theoretical framework for cumulative lifetime risk and its role in accurate diagnosis (Section 3.3); (iii) together with Section 2.3, we will have a unified theoretical framework for identifying a genetic disease by understanding the relationships between *D*, *G*, and *T* as summarized in Section 5.

**Necessary diagnostic tests**. An essential property of a diagnostic test is that it be effective at detecting the disease when the test is administered to an individual having the disease. More precisely, it should be the situation that P(T|D)≈1; otherwise, this particular test should not be used as a diagnostic tool. Sometimes, P(T|D) is referred to as the *true-positive rate*, as well as the *sensitivity* of the diagnostic test [25].

Recall that P(T|D)=1 is equivalent to saying that *T* is *necessary* for *D* (details of the equivalency are in Section 2.3 with *G* replaced by *T*); that is, “*T* is necessary for *D*” is equivalent to the diagnostic test having high sensitivity. Similarly, one can show that P(T|D)=1 is equivalent to saying that the false-negative rate is zero ($P(T^{\prime }|D)=0$). Therefore, “*T* is necessary for *D*” (i.e., the diagnostic test has high sensitivity or has a small false-negative rate) means that: if a person has the disease, then the person will almost always test positive for the disease. When *T* is necessary for *D*, the population is partitioned, as shown in Table 1 with $n_{12}\approx 0$:

**Sufficient diagnostic tests**. A diagnostic test becomes a useful way of identifying those with the disease if P(D|T)≈1. Sometimes, P(D|T) is referred to as the *positive predictive rate* [25].

Recall that P(D|T)=1 is equivalent to saying that *T* is *sufficient* for *D* (details of the equivalency are in Section 2.3 with *G* replaced by *T*); that is, “*T* is sufficient for *D*” is equivalent to the diagnostic test having a high positive predictive rate. Similarly (assuming $P(D^{\prime })\neq 0$), one can show that P(D|T)=1 is equivalent to saying that: the false-positive rate is zero ($P(T|D^{\prime })=0$); as well as $P(T^{\prime }|D^{\prime })=1$. Sometimes, $P(T^{\prime }|D^{\prime })$ is called the *true-negative rate*, as well as the *specificity* of the diagnostic test [25]. Therefore, “*T* is sufficient for *D*” (i.e., the diagnostic test has a high positive predictive rate or a small false-positive rate, or high specificity) means that: if a person receives a positive test, then the person will almost always have the disease. When *T* is sufficient for *D*, the population is partitioned, as shown in Table 1 with $n_{21}\approx 0$:

**Accurate diagnosis: A necessary and sufficient diagnostic test**. The goal of any diagnostic test is for a positive test result to be *both* necessary and sufficient for an individual to be identified with the disease; that is, *T* and *D* partition the population as a *diagonal partition* (Table 1 with $n_{12}\approx 0$ and $n_{21}\approx 0$), and those individuals in the population under consideration with the disease are *precisely* those individuals who receive a positive result from the diagnostic test. Only if *both* sensitivity and specificity are high in a clinical study can clinicians be confident their analyses are accurate.

In summary, the result of the foregoing is that accurate diagnosis depends on *T* being both necessary and sufficient for *D*. When this is the case, P(T)=P(D). Thus, an estimator for P(T) based on a clinical study should be close to an estimator for P(D) described by Equation (3).

An implication for clinicians is that if they choose to use a diagnostic test with a positive test result being “not necessary” for the occurrence of the disease, then that is equivalent to them accepting a significant iceberg effect and a large underestimation of the actual prevalence of the disease. Another implication for clinicians is that if they believe a diagnostic test’s positive test result is “necessary, but not sufficient” for the occurrence of the disease, then that is equivalent to them accepting that the diagnostic test does not accurately predict whether a person has the disease or not. Instead, we suggest that it is imperative that clinicians continue their investigations—ultimately seeking a diagnostic test that *does* yield P(T)=P(D).

#### 3.2.2. Estimating Prevalence via a Diagnostic Test

To actually create a diagnostic test that yields P(T)≈P(D), a clinician should begin with a diagnostic test for which *T* is *necessary* for *D* (Table 1 with $n_{12}\approx 0$). Indeed, if *T* is not necessary for *D*, then the diagnostic procedure ought to be rejected outright. When diagnostic tests are first developed, they are likely to have difficulty identifying those with the disease and those without it (Table 1 with $n_{21}\neg \approx 0$ and, therefore, $n_{11}$ is underestimated). A clinician’s goal is therefore to refine the diagnostic test, while keeping in mind accepted clinical study design protocols [7], so that it also ensures *T* is *sufficient* for *D* (Table 1 with $n_{12}\approx 0$ and $n_{21}\approx 0$). When this is achieved, clinicians will have created a diagnostic test that accurately predicts disease presence (i.e., the test is ready for usage as a diagnostic tool), and P(T) will be close to P(D).

The preceding intuitive discussion connects our theory to a clinician’s practice. To our knowledge, we are the first to rigorously characterize the discussion by developing the theoretical mechanism for *how* P(T) approaches P(D) as the diagnostic test is refined. We demonstrate that when *T* is necessary for *D* (Section 3.2.1), P(T) can be used to provide lower and upper bounds for P(D); moreover, we show that as the false-positive rate ($P(T|D^{\prime })$) approaches zero, the lower and upper bounds force P(T) to approach P(D). Thus, *T* will be both necessary and sufficient for *D*, and consequently, P(T)≈P(D). Specifically, the theoretical mechanism is described by

(4) $$P\left(T\right)-\left(1-P\left(T\right)\right)\frac{\alpha_{0}}{1-\alpha_{0}}\leq P\left(D\right)\leq P\left(T\right)\;,$$

where $\alpha_{0}$ is an upper bound for $P(T|D^{\prime })$; in other words, the false-positive rate is at most $\alpha_{0}$ ($0\leq P\left(T|D^{\prime }\right)\leq \alpha_{0}$). The derivation of Equation (4) is provided in Appendix C.

Reducing $\alpha_{0}$ improves the diagnostic test’s accuracy. Moreover, Equation (4) describes the theoretical mechanism by which P(T) approaches P(D) as $\alpha_{0}$ becomes smaller (because the lower bound in Equation (4) approaches P(T) as $\alpha_{0}$ approaches zero), resulting in the partition of the population induced by *T* and *D* approaching a diagonal partition, at which point, *T* will be both necessary and sufficient for *D*. The implication is crucial:

As the false-positive rate becomes smaller, the probability increases that a positive result in the corresponding diagnostic test will more accurately predict prevalence of the disease.

**Estimation procedure**. The above theoretical development suggests the following four-step procedure for clinicians wanting to use a diagnostic test to accurately estimate disease prevalence:

(i) Begin with a diagnostic test for which *T* is necessary for *D*. A corresponding clinical study should consist of data resembling Table 1 with $n_{12}\approx 0$.
(ii) Estimate P(T). Use Table 1 to find
  $$\hat{P}\left(T\right)=\frac{n_{11}+n_{21}}{n}\;\cdot$$
(iii) Estimate the maximum value of a false-positive rate, which is denoted by $\alpha_{0}$. Use Table 1 to compute, for example, a 95% confidence interval [5,6] for the false-positive rate, and take $\alpha_{0}$ to be the maximum of the interval
  $$\alpha_{0}=\hat{\alpha }+1.96\sqrt{\hat{\alpha }\left(1-\hat{\alpha }\;\right)/n}\;,\;\mathrm{where}\;\hat{\alpha }=\hat{P}\left(T|D^{\prime }\right)=\frac{n_{21}}{n_{21}+n_{22}}\;\cdot$$
(iv) Substitute the estimators of P(T) and $\alpha_{0}$ into Equation (4), which yields an interval estimate for P(D).

**Figure 3:**
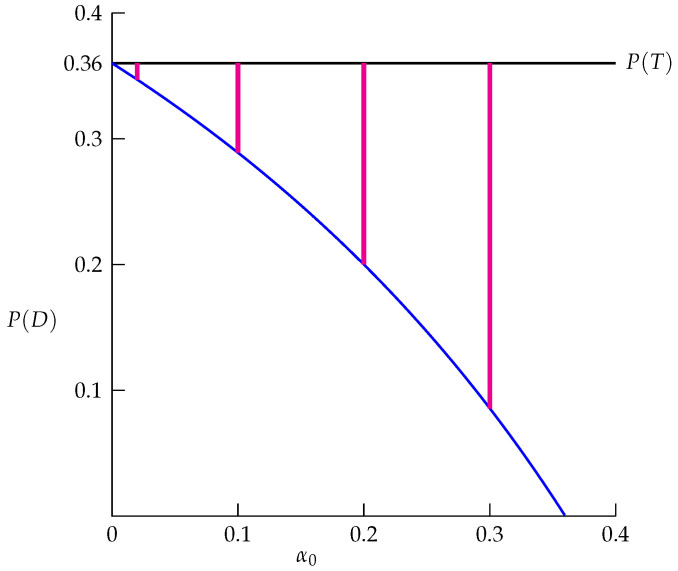
Illustration of Example 1, where P(T)=0.36. The horizontal axis is the values for $\alpha_{0}$, which is an upper bound for the false-positive rate; the vertical axis is the disease prevalence P(D). The lower bound in Equation (4) is the blue curve, and the upper bound is the black horizontal line at P(T)=0.36. P(D) will lie inclusively between the two bounds. The interval estimates for P(D) (indicated in red) are shown for $\alpha_{0}=0.3$, 0.2, 0.1, and 0.02. The accuracy of an interval estimate of disease prevalence increases as the false-positive rate declines; specifically, the red interval estimates become smaller as $\alpha_{0}$ becomes smaller.

**Example** **1.** *As context, consider a disease caused by a dominant allele with p=0.2, r=1 and the genotype CC fully penetrant. Then P(D)=0.36 (Equation (3)). In principle, an accurate diagnostic test should yield P(T)≈P(D)≈0.36. To achieve this, begin with a diagnostic test for which T is necessary for D (Step (i)). Using a corresponding clinical study resembling Table 1 with $n_{12}\approx 0$, obtain the estimator $\hat{P}\left(T\right)\approx 0.36$ (Step (ii)). Figure 3 is an illustration of Equation (4), where the lower bound is the blue curve and the upper bound is the black horizontal line (at $\hat{P}\left(T\right)\approx 0.36$). The disease prevalence P(D) lies inclusively between the two bounds, and interval estimates for P(D) (indicated in red) are shown for $\alpha_{0}=0.3$, 0.2, 0.1, and 0.02. Depending on the diagnostic test and how it is interpreted, false-positive results may generate uncertainty regarding P(D); for example, if the false-positive rate is as high as 0.3 (i.e., $\alpha_{0}=0.3$), then P(D) is estimated as being inclusively between 0.086 and 0.36 (Steps (iii) and (iv); Figure 3). An interval estimate with such a large spread makes any P(D) estimate unreliable (e.g., the interval does not support claiming P(D)≈0.09). Indeed, such uncertainty should alert clinicians that the diagnostic test is not accurate (T is necessary, but not yet sufficient for D). However, as $\alpha_{0}$ is reduced, the test’s accuracy is improved; at values $\alpha_{0}\leq 0.1$, the disease prevalence will be estimated more accurately (Figure 3 with α=0.1 and 0.02); T will become both necessary and sufficient for D, resulting in P(T)≈P(D)≈0.36, as desired.*

Incidentally, our development of accurate diagnosis applies to *any* disease, whether it is genetically based or not.

### 3.3. Accurate Diagnosis Requires Cumulative Lifetime Risk

For many disorders, disease prevalence is a cumulative lifetime risk; that is to say, disease prevalence is the likelihood a person from the population will be accurately diagnosed as having the disease at some point during their lifetime. For certain disorders, in particular those caused by dominant alleles, symptoms and the probability of testing positive for the disease (P(T)) show a peak in middle age. This leads to a steady accumulation of cases (of a particular disease) in the population [8,11,13,26,27]. Diagnostic tests for such diseases are administered to people thought to have the disease-causing genotype; these tests yield a result at a specific moment in each person’s lifetime. For some disorders (e.g., Huntington’s Disease (HD) [28]), the probability of a positive test result (P(T)) increases with age, so young people with the disease-causing genotype may not test positive for the disease. In non-fatal dominant diseases, these negative results are often misinterpreted to mean that such people will never test positive for the disease. Our analysis will make clear that this interpretation is unwarranted and is a source of underestimates of P(D).

**Figure 4:**
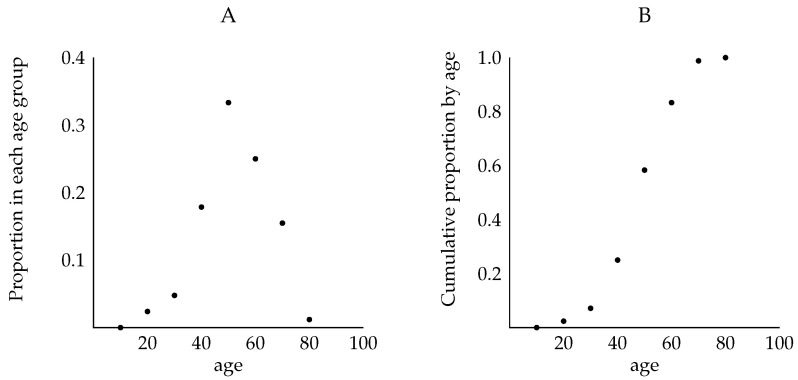
The cumulative lifetime feature of disease prevalence for people with Huntington’s Disease (HD). (**A**) Proportion of 84 people (ranging in age from 10 to 80) with HD who are diagnosed at each of the eight age decades. The maximum proportion occurs at approximately age 50, and the distribution is bell-shaped, but not symmetric. (**B**) The cumulative proportion of the people shown in (**A**) with HD who are diagnosed at each of the eight decades. Constructed from data in [28].

Figure 4 shows the cumulative lifetime feature of disease prevalence for people with HD. Figure 4A illustrates data for 84 people (ranging in age from 10 to over 80 years old) who at some point developed HD. The maximum proportion was diagnosed at approximately age 50, and by age 80 nearly all of those who would develop HD had been diagnosed. Figure 4B illustrates the corresponding cumulative distribution of diagnosis, indicating that it takes about 80 years for most people with the disease-causing genotype for HD to be identified. This cumulative mechanism means that a negative diagnostic test result at any age below, say 70, does not preclude either a positive diagnostic test result or actual disease itself at a later time. Therefore, HD prevalence cannot be accurately estimated by studying only those younger than age 70. This cumulative pattern of diagnosis applies to prion diseases [13] and amyotrophic lateral sclerosis [29], and in general has implications for the estimation of the prevalence of diseases that are detected only later in life.

Genetic tests at any time will show the presence or absence of the disease-causing genotypes. For a disease such as HD, the CC genotype is unlikely to be found in living people because most individuals with the CC genotype die before birth. The presence of the Cc genotype suggests that the disease will develop in severity over the lifetime of the individual and the true prevalence P(D) is not accurately estimated until all ages have been accounted for [13]. Thus, for individuals with the Cc genotype, the variable appearance of HD over a lifespan is not necessarily a measure of the penetrance of the disease-causing genotype Cc, as disease prevalence may also depend on how carefully clinicians have diagnosed the condition (i.e., how likely it is to obtain a positive diagnostic test result may depend on disease severity and the diagnostic test’s ability to detect mild forms of the disease).

Cumulative lifetime risk is best understood as an investigation of the accuracy of diagnosis and the identification of all people who might have the disease. Recall that an accurate diagnosis can be framed in terms of a positive diagnostic test result being both necessary and sufficient for the presence of the disease (Section 3.2.1). The implications are crucial for understanding population disease prevalence. We will show that cumulative lifetime risk is *formally and actually* equal to population-wide disease prevalence, P(D):

**Theoretical framework**. The following is a theoretical framework for cumulative lifetime risk analysis. It describes the accuracy of a diagnosis as a function of subject age in terms of two measures of cumulative diagnosis, which we call the *cumulative age-true positive rate* and the *cumulative age-positive predictive rate*. The former is an index of the diagnostic test’s true-positive rate, and thus of the degree to which the diagnostic test is *necessary* for demonstrating the disease; the latter is an index of the diagnostic test’s positive predictive rate, and thus of the degree to which the diagnostic test is *sufficient* for demonstrating the disease. For simplicity, we assume that the maximum lifetime of individuals in the population is 100 years.

We define the *age-true positive rate*, denoted by $f_{tpr}\left(i\right)$, to be the conditional probability a person receives a positive test result at age *i* years old (i=1,2,…,100), given the person has the disease; that is to say,

$$f_{tpr}\left(i\right)=P\left(\left(T\cap \left\{\mathrm{age}\;i\;\mathrm{years}\;\mathrm{old}\right\}\right)|D\right)\;\left(i=1,\;2,\ldots ,100\right).$$

Thus, the true-positive rate is the accumulation of all age-true positive rates,

$$P\left(T|D\right)=\sum_{i=1}^{100}f_{tpr}\left(i\right)\;.$$

We define the *age-positive predictive rate*, denoted by $f_{ppr}\left(i\right)$, to be the conditional probability a person has the disease at age *i* years old, given the person receives a positive test result (i=1,2,…,100); that is to say,

$$f_{ppr}\left(i\right)=P\left(\left(D\cap \left\{\mathrm{age}\;i\;\mathrm{years}\;\mathrm{old}\right\}\right)|T\right)\;\left(i=1,\;2,\ldots ,100\right).$$

Thus, the positive predictive rate is the accumulation of all age-positive predictive rates,

$$P\left(D|T\right)=\sum_{i=1}^{100}f_{ppr}\left(i\right)\;.$$

Here are the properties that both the age-true positive rate and the age-positive predictive rate satisfy (to simplify the notation, the function f(i) stands for both $f_{tpr}\left(i\right)$ and $f_{ppr}\left(i\right)$):

(i) The function f(i) has values 0≤f(i)≤1 for all i=1,2,…,100.
(ii) The sum of all the values of f(i) must equal one, $\sum_{i=1}^{100}f\left(i\right)=1$, which is a consequence of the diagnostic test satisfying P(T|D)=1 (*T* is necessary for *D*) and P(D|T)=1 (*T* is sufficient for *D*).
(iii) The function f(i) is bell-shaped, but is not necessarily symmetric. That is, f(i) obtains its maximum at some age denoted by *m*; f(i) will be an increasing function for i<m and a decreasing function for i>m. For diseases with later-in-life detection (e.g., many diseases caused by dominant alleles), *m* typically occurs during middle-age.

**Figure 5:**
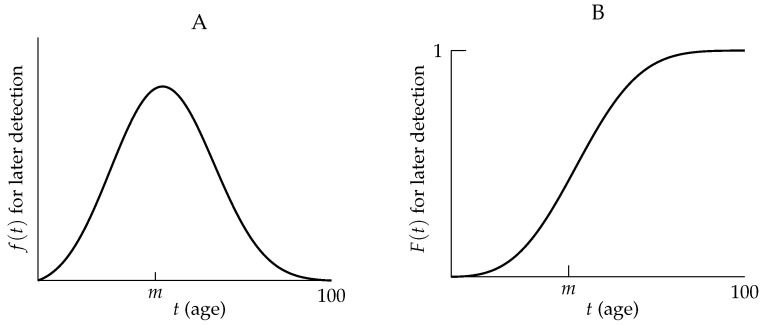
(**A**) Graph of a typical *f*, which stands for both the age-true positive rate ($f_{tpr}$) and the age-positive predictive rate ($f_{ppr}$). See the text for their descriptions. The function f(t) is bell-shaped, but is not necessarily symmetric, and obtains its maximum at some age denoted by *m*. For a disease with later-in-life detection, *m* typically occurs during middle-age. (**B**) Graph of a typical *F*, which stands for both the cumulative age-true positive rate ($F_{tpr}$) and the cumulative age-positive predictive rate ($F_{ppr}$). See the text for their descriptions. For a disease with later-in-life detection, *F* is close to one only after middle age.

Figure 5A provides a graph of a typical *f* (which stands for both $f_{tpr}$ and $f_{ppr}$) for diseases with later-in-life detection. For convenience, the function *f* has been extended to a continuous function defined for all times 0≤t≤100. Indeed, the function f(t) can be thought of as a “best fit curve” using the values f(i) for i=1,2…,100, and f(0)=0.

We define the *cumulative age-true positive rate* of the disease at age *i*, denoted by $F_{tpr}\left(i\right)$, to be the sum of the age-true positive rates for ages at most *i*; that is to say,

$$F_{tpr}\left(i\right)=\sum_{k=1}^{i}f_{tpr}\left(k\right)\;\left(i=1,\;2,\cdots ,100\right)\;.$$

We define the *cumulative age-positive predictive rate* of the disease at age *i*, denoted by $F_{ppr}\left(i\right)$, to be the sum of the age-positive predictive rate for ages at most *i*; that is to say,

$$F_{ppr}\left(i\right)=\sum_{k=1}^{i}f_{ppr}\left(k\right)\;\left(i=1,\;2,\cdots ,100\right)\;.$$

Here are properties that both the cumulative age-true positive rate and the cumulative age-positive predictive rate satisfy (to simplify the notation, the function F(i) stands for both $F_{tpr}\left(i\right)$ and $F_{ppr}\left(i\right)$):

(i) The function *F* has values 0≤F(i)≤1 for the ages i=1,2,⋯,100.
(ii) F(i) is an increasing function, where F(100)=1 because
  $$F_{tpr}\left(100\right)=P\left(T|D\right)=1\;\mathrm{and}\;F_{ppr}\left(100\right)=P\left(D|T\right)=1.$$
(iii) F(i) will be concave up (increasing at an increasing rate) for 1≤i<m; and will be concave down (increasing at a decreasing rate) for m<i≤100.

Figure 5B provides a graph of a typical *F* (which stands for both $F_{tpr}$ and $F_{ppr}$) for diseases with later-in-life detection. For convenience, the function *F* has been extended to a continuous function defined for all times 0≤t≤100. Indeed, the function F(t) can be thought of as a “best fit curve” using the values F(i) for i=1,2,⋯,100, and F(0)=0.

In summary, accurate diagnosis (Section 3.2) in the context of a cumulative lifetime risk corresponds to

$$F_{tpr}\left(100\right)=1\;\mathrm{and}\;F_{ppr}\left(100\right)=1.$$

Framing accurate diagnosis as a cumulative lifetime risk has implications for clinicians regarding a diagnostic test’s result. For diseases with later-in-life detection (e.g., many diseases caused by dominant alleles), clinicians should be aware of three important and related concepts:

(i) A negative diagnostic test result up to middle age does not indicate that the person will never be accurately diagnosed with the disease during their lifetime. For example, a person may actually have an early form of the disease that is not detected by the diagnostic test; consequently, inadequate testing may prevent treatment for the person during their lifetime. Indeed, because $F_{tpr}\left(t\right)\approx 1$ and $F_{ppr}\left(t\right)\approx 1$ only later in life, it is essential to continue testing a person with the disease-causing genotype who receives a negative diagnostic test result well beyond middle age (Figure 5).
(ii) Clinical studies exclusively using people from a specific age group (e.g., only those from 20–30 years old) will suffer from ascertainment bias; hence, such studies will *not* produce meaningful inferences regarding population disease prevalence (Figure 5). Moreover, clinical studies consisting of people only up to middle age will suffer from ascertainment bias and result in an underestimation of the prevalence of diseases with later-in-life detection. For example, HD prevalence would be underestimated by about 30% if only people up to age 55 were included in the data in [28] (Figure 4B).
(iii) A positive diagnostic test result at any age (in a person with the disease-causing genotype) may also be a false-positive and may suggest treatments that will not be necessary. The chances of false positives should thus be minimized at all ages (Figure 5).

**Cumulative lifetime risk equals disease prevalence**. We now show that cumulative lifetime risk in principle equals the prevalence of the disease, P(D) (Section 2). For a diagnostic test in which a positive test result is both necessary and sufficient for the presence of the disease,

Cumulativelifetimerisk=P(T),

as well as

$$P\left(T|D\right)=F_{tpr}\left(100\right)=1\;\mathrm{and}\;P\left(D|T\right)=F_{ppr}\left(100\right)=1.$$

Now,

$$\begin{array}{cc} P(D) & =P\left(D\cap T\right)+P\left(D\cap T^{\prime }\right)\\ P(D) & =P\left(T|D\right)P\left(D\right)+P\left(T^{\prime }|D\right)P\left(D\right)\\ 1 & =P\left(T|D\right)+P\left(T^{\prime }|D\right), \end{array}$$

which implies $P(T^{\prime }|D)=0$ because P(T|D)=1. Thus,

$$\begin{array}{cc} P(D) & =P\left(D\cap T\right)+P\left(D\cap T^{\prime }\right)\\ P(D) & =P\left(D|T\right)P\left(T\right)+P\left(T^{\prime }|D\right)P\left(D\right), \end{array}$$

which implies P(D)=P(T) because P(D|T)=1 and $P(T^{\prime }|D)=0$. Therefore,

Cumulativelifetimerisk=P(T)=P(D),

where P(D) is given by Equation (3).

In summary, it is important to view the accuracy of diagnosis as a function of subject age in order to ensure that a positive diagnostic test result precisely identifies those individuals who have the disease. That is, the goal of any diagnostic test should be for P(T) to accurately estimate P(D).

## 4. Familial and Offspring-Group Aggregation

The current approach to investigating the prevalence of genetic diseases in various families relies on the concept of *familial aggregation*, in which the frequency of a disease may be higher in particular family groupings than in the general population. An initial grouping was the *hereditary family*, consisting of genetic relatives from the same family tree: grandparents, parents, siblings, cousins, etc. [8,11]. A more precise grouping is *first-degree relatives* (parents, offspring, and siblings [30]), which form a subset of the hereditary family. However, a person’s genetic disease risk is not directly influenced by a non-parent in a hereditary family. Because current approaches assess a person’s genetic disease risk via imprecise measures of familial aggregation, we propose they be replaced by a measure determined solely by parental genotypes; thus, we introduce a *new* approach that we call *offspring-group aggregation*. The advantages of this approach will become apparent below.

Throughout, we use standard human pedigree analysis terminology; for example, “parents” refers to genetic parents, and “siblings” refers to offspring with the same genetic parents [8].

**Offspring-groups**. Consider a two-allele model for a genetic disease. Table 2 illustrates all possible parental genotypes and their offspring. The entries in the individual cells are the frequencies of the corresponding offspring.

Constructing all the possible matings using the parents in Table 2, we observe that there are precisely six partition subsets of the general population, which we denote by $F_{i}$ (for i=1, 2, …, 6), and have the following probabilities:

(5) $$\begin{array}{cccc} \; & F_{1}:CC\times CC; & P(F_{1}) & =p^{2}\times p^{2};\\ \; & F_{2}:CC\times Cc; & P(F_{2}) & =4(p^{2}\times pq);\\ \; & F_{3}:CC\times cc; & P(F_{3}) & =2(p^{2}\times q^{2});\\ \; & F_{4}:Cc\times Cc; & P(F_{4}) & =4(pq\times pq);\\ \; & F_{5}:Cc\times cc; & P(F_{5}) & =4(pq\times q^{2});\\ \; & F_{6}:cc\times cc; & P(F_{6}) & =q^{2}\times q^{2}. \end{array}$$

**Figure 6:**
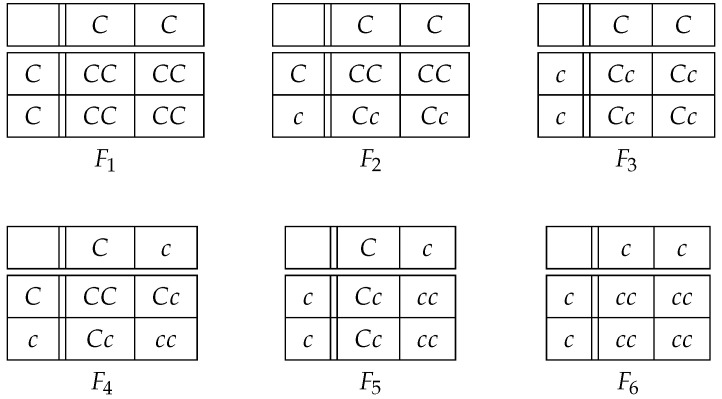
Illustration of the possible offspring genotypes within each of the six offspring-groups $F_{i}$ (for i=1, 2, …, 6). Because offspring genotype frequencies differ among the offspring-groups, some office-spring groups may have high disease prevalence while others may have low or zero disease prevalence.

In Figure 6, we illustrate the possible offspring genotypes within each subset $F_{i}$ (for i=1, 2, …, 6). We refer to $F_{i}$ as an *offspring-group*, which consists of all people (offspring) whose parents have the genotypes that determine the partition $F_{i}$. For example, $F_{2}$ consists of all people (offspring) in the general population whose parents have genotypes CC×Cc.

Consequently, because a person’s genotype is dependent on their parents, siblings belong to the same offspring-group. Moreover, an offspring-group will include people who are not necessarily siblings; indeed, two people who are not siblings could each have parents with the same genotypes and thus be members of the same offspring-group.

Incidentally, which offspring-group a parent belongs to is determined by the genotypes of *their* parents; a parent might not belong to the same offspring-group as their children. For example, suppose you and your mate have genotypes CC×Cc, then your offspring belong to $F_{2}$; in addition, suppose your parents have genotypes Cc×Cc, then you belong to $F_{4}$.

At any given time, there are always exactly six offspring-groups in the general population (Figure 6), while there are a large number of hereditary families with various compositions. Most importantly, Figure 6 shows that some offspring-groups may have high P(D), while others may have low or zero P(D).

Clinical studies involving pairs of siblings report the likelihood that a sibling has the disease, given the other sibling has the disease. This statistic, called sibling risk, is presented as if it were a clinical characteristic of the disease. Disease risk is instead determined by the structures of the offspring-groups (Figure 6), the penetrance of disease-causing genotypes, and the frequency of the disease-causing genotypes. We will address this idea in Section 4.2. Familial aggregation is currently measured with the sibling recurrence-risk ratio, denoted by $\lambda_{s}$, which refers to the ratio of sibling risk to the population-wide disease prevalence (Section 4.1). An estimated high value ${\hat{\lambda }}_{s}\gg 1$ (e.g., occasionally obtained from clinical studies) is used often as an indication that a particular disorder has familial aggregation [10,11,31]. However, as we will show, the current measure of familial aggregation is biased because it ignores a large part of the population and because it is affected by (often mistaken) estimates of population disease prevalence. Indeed, we provide several arguments that, in principle, the theoretical sibling recurrence-risk ratio is always equal to one ($\lambda_{s}$ = 1); this gives the surprising result that any estimator ${\hat{\lambda }}_{s}≉1$ be viewed with suspicion. Therefore, we propose that $\lambda_{s}$ is in need of replacement.

Our new concept focuses on the six offspring-groups (Figure 6) instead of hereditary families. Because each offspring-group has its own disease risk, “familial risk” should not be represented by a population parameter with a single value such as $\lambda_{s}$. After demonstrating the unsuitability of $\lambda_{s}$, we propose an alternative that depends on the allele frequency and penetrance of disease-causing genotypes; thus, our measure differs among the possible six offspring-groups of the general population (Equation (9)). We also discuss why our new measure is likely to yield an unbiased estimator based on clinical studies—unlike estimators for the sibling recurrence-risk ratio (Section 4.2).

### 4.1. Sibling Recurrence-Risk Ratio

*Sibling risk* is defined as the probability that an individual has a disease, given that a sibling has the same disease [11,32,33]. More precisely, let $S_{1}$ and $S_{2}$ denote two (non-identical) siblings with the same parents, let $D_{1}$ denote the event that $S_{1}$ has the disease, and let $D_{2}$ denote the event that $S_{2}$ has the same disease. In the literature [10,11,33], sibling risk is often denoted by $K_{s}$; thus,

$$K_{s}=\mathrm{Sibling}\;\mathrm{risk}=P\left(D_{2}|D_{1}\right).$$

In addition, the population risk (frequency, prevalence, probability) of the disease in the population is often denoted by *K*. In particular, $P(D_{1})=K$ and $P(D_{2})=K$. The literature in this field [10,11] defines the *sibling recurrence-risk ratio*

$$\lambda_{s}=\frac{K_{s}}{K}$$

for use in the explanation of familial aggregation, as well as for hypothesizing a need for additional genes to describe the dependence of disease prevalence on genotype. Misunderstanding and different interpretations of the definition of $K_{s}$ have led to various approaches for (inaccurately) estimating $\lambda_{s}$, making valid inferences and hypotheses problematic [32].

Our approach to this issue is based on the alleles of offspring being dependent on their parents, as well as on the small number of possible offspring-group types in a population and the membership of two siblings in the same offspring-group. Observe that while the siblings $S_{1}$ and $S_{2}$ are from the same offspring-group, the definition of $K_{s}$ as currently used does not specify to *which* of the six offspring-groups the siblings belong (Figure 6). Thus, $K_{s}$ is not defined as a conditional probability with respect to an offspring-group, forcing the general population to become the focus for determining $K_{s}$. Therefore, the heterogeneity of offspring-groups means $\lambda_{s}$ is not an enlightening measure of familial aggregation. Our analysis develops several biologically based probabilistic arguments leading to the demonstration that $K_{s}=K$ for a genetic disease; that is, $\lambda_{s}=1$ (Section 4.1.1 and Section 4.1.2).

Following this demonstration, we will explore its implications for the calculations of estimators for $K_{s}$ and *K*. We also discuss why the estimator ${\hat{\lambda }}_{s}$ experiences computational deficiencies—incorrectly predicting $\lambda_{s}>1$. In addition, we discuss the implications of $K_{s}=K$ and the misuse of $\lambda_{s}$ as the justification for additional gene hypotheses (Section 4.1.3).

#### 4.1.1. Offspring Allele Independence: $\lambda_{s}=1$

The genotypes of offspring are dependent on the parents, not on the siblings; consequently, whether $S_{1}$ has a particular allele is *not* affected by whether $S_{2}$ has the allele and genetic events regarding $S_{1}$ and $S_{2}$ will be independent of each other. In particular, with respect to genetic diseases, $D_{1}$ and $D_{2}$ are independent events. Therefore, $P\left(D_{1}\cap D_{2}\right)=P\left(D_{1}\right)P\left(D_{2}\right)$, which implies

$$K_{s}=P\left(D_{2}|D_{1}\right)=\frac{P(D_{1}\cap D_{2})}{P(D_{1})}=\frac{P\left(D_{1}\right)P\left(D_{2}\right)}{P(D_{1})}=P\left(D_{2}\right)=K\;;$$

hence, we conclude that $\lambda_{s}=1$. This means that $\lambda_{s}=1$ for *any* disease in which disease status is independent in each sibling. Incidentally, the independence of $D_{1}$ and $D_{2}$ may not be the case for certain types of disorders; for example, two siblings living in the same household will likely not be independent of each other with respect to non-genetic contagious disease status [32].

As another approach showing $\lambda_{s}=1$, we note that Risch [33] writes $\lambda_{s}$ in terms of the covariance between siblings

$$\lambda_{s}=1+\frac{1}{K^{2}}\mathrm{Cov}\left(D_{1},D_{2}\right).$$

Because $D_{1}$ and $D_{2}$ are independent events, $\mathrm{Cov}(D_{1},D_{2})=0$ [5,6] and we again conclude that $\lambda_{s}=1$.

As a third approach showing $\lambda_{s}=1$, we note Risch [11] defines $\phi_{s}$ as the probability that two siblings share zero marker alleles and states that $\phi {}_{s}=1/4$. Let $Z=\{S_{1}\;\mathrm{and}\;S_{2}\;\mathrm{share}\;\mathrm{zero}\;\mathrm{alleles}\}$, and observe that

$$P\left(Z\right)=\phi_{s}=1/4.$$

Recall $\left\{S_{1}\;\mathrm{and}\;S_{2}\;\mathrm{have}\;\mathrm{the}\;\mathrm{disease}\right\}=D_{1}\cap D_{2}$. As indicated in [11],

$$P\left(\left(D_{1}\cap D_{2}\right)|Z\right)=P\left(D_{1}\right)P\left(D_{2}\right)=K^{2},$$

which implies

$$P\left(\left(D_{1}\cap D_{2}\right)\cap Z\right)=P\left(\left(D_{1}\cap D_{2}\right)|Z\right)P\left(Z\right)=K^{2}\phi_{s}\;;$$

moreover, $P\left(D_{1}\cap D_{2}\right)=P\left(D_{2}|D_{1}\right)P\left(D_{1}\right)=K_{s}K$. Therefore,

$$P\left(Z|\left(D_{1}\cap D_{2}\right)\right)=\frac{P(\left(D_{1}\cap D_{2}\right)\cap Z)}{P(D_{1}\cap D_{2})}=\frac{K^{2}\phi_{s}}{K_{s}K}=\frac{\phi_{s}}{K_{s}/K}=\frac{\phi_{s}}{\lambda_{s}}.$$

As described in [10], the expected proportion of affected sibling pairs sharing zero alleles is 0.25; that is, $P\left(Z|\left(D_{1}\cap D_{2}\right)\right)=0.25=\phi_{s}$. Hence, $\phi_{s}=\phi_{s}/\lambda_{s}$, and we again conclude that $\lambda_{s}=1$.

#### 4.1.2. Siblings Are from the Same Offspring-Group: $\lambda_{s}=1$

We define the *offspring-group risk* for a specific offspring-group $F_{i}$ to be the probability of an individual having the disease, given that the individual is an offspring in $F_{i}$. That is, offspring-group risk is $P(D|F_{i})$ (for i=1, 2, …, 6).

From Figure 6, using P(D|cc)=0 for a disease *D* caused by a dominant allele (Section 2.2), we compute the offspring-group risk for each of the six offspring-groups:

(6) $$\begin{array}{cccc} \; & P\left(D|F_{1}\right)=P\left(D|CC\right)\;; & P(D|F_{2}) & =\frac{1}{2}\left(P(D|CC)+P(D|Cc)\right)\\ \; & P\left(D|F_{3}\right)=P\left(D|Cc\right)\;; & P(D|F_{4}) & =\frac{1}{4}\left(P(D|CC)+2P(D|Cc)\right)\\ \; & P\left(D|F_{5}\right)=\frac{1}{2}P\left(D|Cc\right) & P(D|F_{6}) & =0. \end{array}$$

We are now ready to compute sibling risk using the offspring-group risks. Because the six offspring-groups form a partition of the population and because siblings are from the same offspring-group, we can write

$$\begin{array}{cc} K_{s}=P\left(D_{2}|D_{1}\right) & =\frac{P(D_{2}\cap D_{1})}{P(D_{1})}\\ \; & =\frac{1}{P(D_{1})}\sum_{i=1}^{6}P\left(D_{2}\cap D_{1}\cap F_{i}\right)\\ \; & =\frac{1}{P(D_{1})}\sum_{i=1}^{6}P\left(D_{2}\cap F_{i}\right)P\left(D_{1}\right)\\ \; & =\sum_{i=1}^{5}P\left(D_{2}|F_{i}\right)P\left(F_{i}\right)\;(\mathrm{because}\;P(D_{2}|F_{6})=0). \end{array}$$

Using the offspring-group frequencies (Equation (5)), we have that

(7) $$\begin{array}{cc} K_{s}=P\left(D_{2}|F_{1}\right)p^{4} & +P\left(D_{2}|F_{2}\right)4p^{3}q+P\left(D_{2}|F_{3}\right)2p^{2}q^{2}\\ \; & +P\left(D_{2}|F_{4}\right)4p^{2}q^{2}+P\left(D_{2}|F_{5}\right)4pq^{3}. \end{array}$$

Substituting the offspring-group risks (Equation (6)) into Equation (7) gives the following representation

$$\begin{array}{cc} K_{s}=P\left(D_{2}|CC\right)p^{4} & +2\left[P\left(D_{2}|CC\right)+P\left(D_{2}|Cc\right)\right]p^{3}q+2P\left(D_{2}|Cc\right)p^{2}q^{2}\\ \; & +\left[P\left(D_{2}|CC\right)+2P\left(D_{2}|Cc\right)\right]p^{2}q^{2}+2P\left(D_{2}|Cc\right)pq^{3}. \end{array}$$

Finally, combining similar terms (and noting that p+q=1), using Equation (1) and Section 2.2, and using Equation (3) yields

$$\begin{array}{cc} K_{s} & =\left[p^{4}+2p^{3}q+p^{2}q^{2}\right]P\left(D_{2}|CC\right)+\left[2p^{3}q+4p^{2}q^{2}+2pq^{3}\right]P\left(D_{2}|Cc\right)\\ \; & =p^{2}\left[p^{2}+2pq+q^{2}\right]P\left(D_{2}|CC\right)+2pq\left[p^{2}+2pq+q^{2}\right]P\left(D_{2}|Cc\right)\\ \; & =p^{2}{\left(p+q\right)}^{2}P\left(D_{2}|CC\right)+2pq{\left(p+q\right)}^{2}P\left(D_{2}|Cc\right)\\ \; & =p^{2}P\left(D_{2}|CC\right)+2pqP\left(D_{2}|Cc\right)\\ \; & =p\left(2r+\left(1-2r\right)p\right)P\left(D_{2}|CC\right)\\ \; & =K. \end{array}$$

Thus, we again conclude that $K_{s}=K$. This last argument has the additional utility that it provides the underlying structure for developing a new measure of aggregation (based on offspring-groups instead of hereditary families), which we discuss in Section 4.2.

Even though the values of $K_{s}$ and *K* are identical, certain offspring-groups (and hereditary families) may have more members with a disease than other groups and may also have a higher or lower P(D) than the population as a whole. The equality of $K_{s}$ and *K* simply means that the sibling recurrence-risk ratio is not an appropriate measure of aggregation among offspring-groups or hereditary families. Before we propose an alternative measure that avoids the challenges associated with $\lambda_{s}$, we discuss why estimators (${\hat{\lambda }}_{s}$) of $\lambda_{s}$ appear to be greater than one.

#### 4.1.3. Estimating the Sibling Recurrence-Risk Ratio

There are two main reasons for errors in the traditional statistical construction of the estimator ${\hat{\lambda }}_{s}$: (i) the prevalence of the disease, *K*, is almost always underestimated; (ii) sibling risk, $K_{s}$, is almost always overestimated.

Having already discussed the underestimation of *K* (Section 3), we now discuss the overestimation of $K_{s}$. Recall that

$$K_{s}=P\left(D_{2}|D_{1}\right)=\frac{P(D_{2}\cap D_{1})}{P(D_{1})}\;\cdot$$

Using data from a clinical study consisting of pairs of siblings, an estimator $\hat{P}\left(D_{2}\cap D_{1}\right)$ will likely yield an overestimation of $P(D_{2}\cap D_{1})$ because the clinical study will almost always not include siblings from offspring-group $F_{6}$ for which $P(D|F_{6})=0$ (Equation (6)). Hence, ascertainment bias will cause

$${\hat{K}}_{s}=\frac{\hat{P}\left(D_{2}\cap D_{1}\right)}{\hat{P}\left(D_{1}\right)}$$

to be overestimated. Incidentally, the contribution of offspring-group $F_{6}$ can be significant. For example, when p≤0.2, more than 40% of all population members are in this offspring-group; thus, the same proportion (more than 40%) of the population is likely not included in computing an estimator for $K_{s}$ (though $F_{6}$ is likely to be included in computing an estimator for *K*).

In addition, we point out that the sibling recurrence-risk ratio is particularly sensitive to underestimates of *K*. Indeed, observe that

$$\lambda_{s}=\frac{K_{s}}{K}=\frac{P(D_{2}|D_{1})}{K}=\frac{P(D_{1}\cap D_{2})}{P(D_{1})K}=\frac{P(D_{1}\cap D_{2})}{K^{2}}.$$

Because the exponent for *K* is two, while $P(D_{1}\cap D_{2})$ has exponent one, $\lambda_{s}$ will be more sensitive to underestimates of *K* than to overestimates of $P(D_{2}\cap D_{1})$.

Similarly, an estimator for $K_{s}$ based on a conditional probability approach is also almost always overestimated. Consider a clinical study consisting of pairs of siblings with one of the siblings known to have the disease. An estimator of $K_{s}$ will be ${\hat{K}}_{s}=\hat{P}\left(D_{2}|D_{1}\right)$. In this case, the clinical study will likely consist mostly of individuals participating from offspring-groups with high offspring-group risks (Equation (6)) [32]; that is, the clinical study will suffer from ascertainment bias. Hence, the calculated value of ${\hat{K}}_{s}$ will likely yield an overestimation of $K_{s}$.

Despite the reality that in principle $K_{s}=K$, several studies [10,11,31,34] have used estimators of $K_{s}$ and *K* derived from clinical studies to suggest $\lambda_{s}>1$ and propose that a more complicated genetic model is required to explain the causes of certain genetic disorders. However, as we have shown that $\lambda_{s}=1$, it appears that equations using $\lambda_{s}$ with a value other than 1 should not be used to propose alternative genetic hypotheses.

As an illustration, we now discuss an example where using $\lambda_{s}$ is problematic. The contribution of the Human Leukocyte Antigen (HLA) region (denoted by $\lambda_{s\mathrm{HLA}}$) to the sibling recurrence-risk ratio is the “expected proportion of affected sibling pairs sharing zero haplotypes identical-by-decent (IBD) (0.25) divided by the observed proportion [of affected sibling pairs sharing zero haplotypes IBD]” [10]; that is,

$$\lambda_{s\mathrm{HLA}}=\frac{P(Z|\left(D_{1}\cap D_{2}\right))}{\hat{P}\left(Z|\left(D_{1}\cap D_{2}\right)\right)}=\frac{0.25}{\hat{P}\left(Z|\left(D_{1}\cap D_{2}\right)\right)}$$

where $Z=\{S_{1}\;\mathrm{and}\;S_{2}\;\mathrm{share}\;\mathrm{zero}\;\mathrm{haplotypes}\}$.

Assuming a multiplicative model [11], the percentage of the HLA’s contribution to the sibling recurrence-risk ratio (denoted by $\%\;\lambda_{s\mathrm{HLA}}$) is calculated [10] using the equation

$$\%\;\lambda_{s\mathrm{HLA}}=100\frac{\log(\lambda_{s\mathrm{HLA}})}{\log(\lambda_{s})}$$

which obviously requires $\lambda_{s}\neq 1$ (otherwise, the denominator is zero). However, because of our earlier discussion that $\lambda_{s}=1$ (Section 4.1.1 and Section 4.1.2), we conclude that this equation experiences a theoretical deficiency by always producing an undefined result—assuming the true value of $\lambda_{s}$ is used.

In addition to the already-discussed issues with the estimator ${\hat{\lambda }}_{s}$, it appears that estimating $\lambda_{s\mathrm{HLA}}$ also is problematic; indeed, the above equation for $\%\;\lambda_{s\mathrm{HLA}}$ often is used with an estimated value of $\lambda_{s}$ satisfying ${\hat{\lambda }}_{s}>1$ and an estimated value of $\lambda_{s\mathrm{HLA}}$ also satisfying ${\hat{\lambda }}_{s\mathrm{HLA}}>1$ [10,11,31,34]. For example, Table 3 in [10] includes several clinical studies that can be used to construct ${\hat{\lambda }}_{s\mathrm{HLA}}$, where the individual studies produce values of $\hat{P}\left(Z|\left(D_{1}\cap D_{2}\right)\right)$ ranging from a low of 0 (also the median and mode) to a high of 0.50. These values correspond to ${\hat{\lambda }}_{s\mathrm{HLA}}$ ranging from undefined (infinite) to 0.50. Combining all of the data in the clinical studies produces $\hat{P}\left(Z|\left(D_{1}\cap D_{2}\right)\right)=0.07$, but due to the large spread of the data, it is not likely that this single value is meaningful (as was pointed out by the authors of the study) [10]. In any event, even if researchers wrongly use ${\hat{\lambda }}_{s}>1$ and ${\hat{\lambda }}_{s\mathrm{HLA}}>1$, they will still be able to compute the quantity

$$\%\;{\hat{\lambda }}_{s\mathrm{HLA}}=100\frac{\log(\;{\hat{\lambda }}_{s\mathrm{HLA}})}{\log(\;{\hat{\lambda }}_{s})}.$$

However, inferences and hypotheses should not be based on such a calculated value of $\%\;{\hat{\lambda }}_{s\mathrm{HLA}}$ because of the previously discussed issues with the estimator ${\hat{\lambda }}_{s}$ and because of difficulties associated with the estimator ${\hat{\lambda }}_{s\mathrm{HLA}}$. We do not dispute that, in principle, there may exist a percentage of HLA’s contribution to disease risk; we are simply proposing that using $\%\;{\hat{\lambda }}_{s\mathrm{HLA}}$ as an indicator is suspect.

In summary, our analysis shows that $\lambda_{s}$ experiences theoretical and computational deficiencies; in addition, its definition often is misunderstood and subject to misinterpretations [32]. These attributes lead to estimators of $\lambda_{s}$ being greatly inflated (${\hat{\lambda }}_{s}\gg 1$); thus, drawing conclusions based on ${\hat{\lambda }}_{s}$ is suspect. In particular, we propose that $\lambda_{s}$ does not accurately indicate familial aggregation nor provide insight for the general genotype–disease relationship.

### 4.2. Offspring-Group Aggregation and Its Measure

To better account for the fact that each offspring-group has its own disease risk, we propose replacing the concept of familial aggregation with what we call *offspring-group aggregation*, which describes the aggregation of genetic diseases among the six offspring-groups (instead of among hereditary families). In addition, we propose a new measure that precisely describes the frequency distribution of genetic diseases among the six offspring-groups and yields estimators of the offspring-group aggregation of genetic diseases.

To do this, we define the *offspring-group recurrence-risk ratio* as the ratio of the offspring-group risk to the disease prevalence; specifically,

$$\mu_{i}=\frac{P(D|F_{i})}{P(D)}\;(\mathrm{for}\;i=1,\;2,\;3,\;4,\;5,\;6).$$

It measures the likelihood that a person from offspring-group $F_{i}$ has the disease, relative to a person from the general population. For example, $\mu_{i}=2.5$ means that a person from $F_{i}$ is about 2.5 times more likely to have the disease as a person from the general population.

Using Equations (1) and (6), we obtain the following representations of offspring-group risk (Section 4.1.2) in terms of *r* and P(D|CC):

$$\begin{array}{cccc} P(D|F_{1}) & =P(D|CC), & P(D|F_{2}) & =\frac{1}{2}\left(1+r\right)P\left(D|CC\right)\\ P(D|F_{3}) & =rP(D|CC), & P(D|F_{4}) & =\frac{1}{4}\left(1+2r\right)P\left(D|CC\right)\\ P(D|F_{5}) & =\frac{1}{2}rP\left(D|CC\right), & P(D|F_{6}) & =0, \end{array}$$

which we collectively write in the form

(8) $$P\left(D|F_{i}\right)=\beta_{i}\left(r\right)P\left(D|CC\right)\;(\mathrm{for}\;i=1,\;2,\;3,\;4,\;5,\;6)$$

where the functions $\beta_{i}\left(r\right)$ are:

$$\begin{array}{cccccc} \beta_{1}\left(r\right) & =1, & \beta_{2}\left(r\right) & =\frac{1}{2}\left(1+r\right), & \beta_{3}\left(r\right) & =r,\\ \beta_{4}\left(r\right) & =\frac{1}{4}\left(1+2r\right), & \beta_{5}\left(r\right) & =\frac{1}{2}r, & \beta_{6}\left(r\right) & =0. \end{array}$$

Using Equations (3) and (8), we obtain

(9) $$\mu_{i}=\frac{\beta_{i}\left(r\right)}{p(2r+(1-2r)p)}\;(\mathrm{for}\;i=1,\;2,\;3,\;4,\;5,\;6).$$

We propose that the values of $\mu_{i}$ are an appropriate way to measure the degree of offspring-group aggregation across all offspring-groups in the general population.

In Table 3, we provide illustrative examples of the offspring-group recurrence-risk ratio (Equation (9)): (i) a *C* allele with p=0.2 and r=1; (ii) a *C* allele with p=0.2 and r=0.5; (iii) a *C* allele with p=0.02 and r=1.

Table 3 illustrates several key features regarding the ability of $\mu_{i}$ to measure offspring-group aggregation:

(i) The disparate values of $\mu_{i}$ show that each offspring-group has its own contribution to offspring-group aggregation. For example, when p=0.2 and r=1, members of offspring-groups $F_{1}$, $F_{2}$, and $F_{3}$ are approximately three-times as likely to have the disease as members of the general population, while family $F_{6}$ will have no members with the disease.
(ii) The distribution of offspring-group aggregation is influenced by the frequency of the dominant allele *C*. For example, when r=1, the positive values of $\mu_{i}$ increase markedly as *p* changes from p=0.2 to p=0.02.
(iii) The distribution of offspring-group aggregation is influenced by the parameter *r*. For example, when p=0.2, the offspring-group aggregation is more concentrated among families $F_{1}$ and $F_{2}$ for r=0.5 than for r=1.

An important property of the values of the offspring-group recurrence-risk ratio $\mu_{i}$ is that their weighted sum is equal to 1, where the individual weights are the frequencies of the corresponding offspring-groups. Indeed, writing Equation (7) in terms of the offspring-group recurrence-risk ratios yields

$$K_{s}=P\left(D_{2}\right)\left[\;p^{4}\mu_{1}+4p^{3}q\mu_{2}+2p^{2}q^{2}\mu_{3}+4p^{2}q^{2}\mu_{4}+4pq^{3}\mu_{5}\right]\;.$$

Recalling that $K=P(D_{2})$, we obtain the following decomposition of the sibling recurrence-risk ratio $\lambda_{s}$ in terms of the offspring-group recurrence-risk ratios $\mu_{i}$

$$\lambda_{s}=\frac{K_{s}}{K}=p^{4}\mu_{1}+4p^{3}q\mu_{2}+2p^{2}q^{2}\mu_{3}+4p^{2}q^{2}\mu_{4}+4pq^{3}\mu_{5}.$$

Because $\lambda_{s}=1$ (Section 4.1.1 and Section 4.1.2), it follows that

(10) $$p^{4}\mu_{1}+4p^{3}q\mu_{2}+2p^{2}q^{2}\mu_{3}+4p^{2}q^{2}\mu_{4}+4pq^{3}\mu_{5}=1\;,$$

where the coefficients of $\mu_{i}$ are the corresponding frequencies of offspring-group $F_{i}$ given by Equation (5).

In addition, another key feature of the offspring-group recurrence-risk ratio is that, unlike $\lambda_{s}$, Equation (10) precisely describes the frequency distribution of offspring-group aggregation of the disease among the six offspring-groups (recall for family $F_{6}$ that $\mu_{6}=0$). Writing Equation (10) in the form

$$\sum_{i=1}^{6}P\left(F_{i}\right)\mu_{i}=1$$

emphasizes that each term in the sum, $P\left(F_{i}\right)\mu_{i}$, is the offspring-group proportion of those with the disease who are in offspring-group $F_{i}$, where $P(F_{i})$ is given by Equation (5).

Table 4 illustrates the offspring-group proportions when p=0.2 and r=1. The implication of the values is straightforward; for example, of those people with the disease, approximately 57% are from offspring-group $F_{5}$. Moreover, notice that the sum of the values equals 1, as required by Equation (10).

We point out that, for diseases in which the genotype CC is lethal prior to birth or shortly thereafter (e.g., Huntington’s disease and Marfan syndrome [35,36]), offspring-groups $F_{1}$, $F_{2}$, and $F_{3}$ will not appear in the (living) population. In this case, the offspring-group risk ratios $\mu_{4}$ and $\mu_{5}$ and the offspring-group proportions $P\left(F_{4}\right)\mu_{4}$ and $P\left(F_{5}\right)\mu_{5}$ are the most relevant.

In summary, our theoretical framework proposes replacing familial aggregation with offspring-group aggregation and replacing $\lambda_{s}$ with the offspring-group recurrence-risk ratio $\mu_{i}$, which has these advantageous properties: (i) it quantifies the clustering of the genetic disease within different offspring-groups and thus does not assume a single value of aggregation that applies across the general population; (ii) it depends on the parameters *p* and *r*, which can be estimated using unbiased clinical studies (Section 2); (iii) unlike $\lambda_{s}$, it does not explicitly depend on *K*, which is often underestimated (Section 3); (iv) it can be used to precisely describe the frequency distribution of offspring-group aggregation (Equation (10)), which cannot be done with $\lambda_{s}$. This emphasizes the importance for parental-sibling clinical studies of determining from which of the six offspring-groups each subject comes.

In Section 5.3, we provide a scenario illustrating how a clinician may use the theoretical framework for offspring-group aggregation as a clinical tool.

## 5. Discussion: Integration of Results

Researchers and clinicians who want to identify a genetic disease, including its genotype-phenotype relationship, benefit from being attentive to the three topics we have developed: (1) the relationship between the disease-causing genotypes and the presence of the associated disease (Section 2); (2) the role of diagnostic tests and their ability to identify the disease (Section 3); and (3) the frequency distribution of offspring-group aggregation among the six offspring-groups (Section 4).

**Figure 7:**
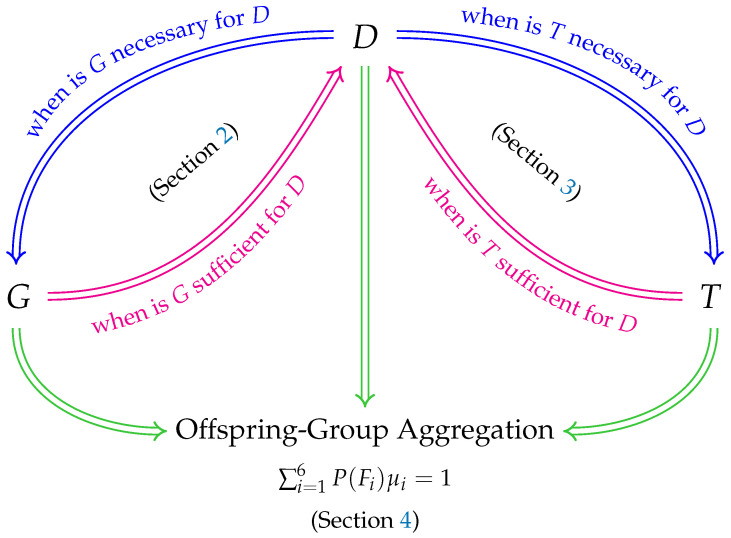
Organizational diagram of our unified theoretical framework of the three main topics for identifying a genetic disease. Recall that *G*, *D*, and *T* each denote the events that an individual from the general population has the disease-causing genotypes, has the disease, and receives a positive test result from a diagnostic test, respectively. The possible relationships between *G*, *D*, and *T* are illustrated by the blue and red arrows (the arrows are the notation for the logical concept “implies”). The frequency distribution of offspring-group aggregation among the six offspring-groups is summarized by the equation, which is affected by *G*, *D*, and *T*, as illustrated by the green arrows.

Figure 7 provides an organizational diagram of our unified theoretical framework of these three topics. Recall that *G*, *D*, and *T* denote the events that an individual from the general population has the disease-causing genotypes, has the disease, and receives a positive test result from a diagnostic test, respectively. Their possible relationships (logical implications) are illustrated by the blue and red arrows: Section 2 discusses when *G* is necessary and/or sufficient for *D* (i.e., when the disease-causing genotypes identify the disease); Section 3 discusses when *T* is necessary and/or sufficient for *D* (i.e., when a diagnostic test identifies the disease). Section 4 investigates the frequency distribution of offspring-group aggregation among the six offspring-groups (summarized by $\sum_{i=1}^{6}P\left(F_{i}\right)\mu_{i}=1$), which is affected by *G*, *D*, and *T*, as indicated by the green arrows.

### 5.1. Relationship between G and D (Section 2)

Fundamental to identifying a genetic disease is determining the relationship between the disease-causing genotypes and the presence of the associated disease. For a disease caused by a dominant allele: *G* is *always* necessary for *D*; *G* is sufficient for *D if and only if* the disease-causing genotypes are fully penetrant. This is illustrated in Figure 7: D⇒G and the corresponding blue arrow *always* occurs; G⇒D and the corresponding red arrow occurs *if and only if* P(D|CC)=1 and P(D|Cc)=1.

In other words, the relationship between disease prevalence and the frequencies of the disease-causing genotypes is always

P(D)≤P(G),

and

P(D)=P(G)onlywhenP(D|CC)=1andP(D|Cc)=1.

The theoretical framework presented in Section 2 provides guidance to researchers and clinicians with regard to determining the relationship between the disease-causing genotypes and the presence of the associated disease. In particular, if they believe “*G* is necessary, but not sufficient for *D*”, then we propose that researchers and clinicians continue their investigations, being aware of the associated consequences and responsibilities (Section 2.3), with the goal of characterizing the relationship between *G* and *D*. Even so, it is essential that clinicians not use their belief that a disease-causing genotype is partially penetrant as justification for using an inaccurate diagnostic test; that is, for using a diagnostic test with low sensitivity and/or low specificity (Section 5.2).

### 5.2. Relationship between T and D (Section 3)

The theoretical framework presented in Section 3 provides guidance to researchers and clinicians with regard to understanding the relationship between a positive diagnostic test result and the presence of the associated disease. In summary, we recommend that researchers and clinicians:

(i) Ensure diagnostic tests have *T* that is both necessary and sufficient for *D*. Figure 7 illustrates the desired relationship: T⇔D and the corresponding blue and red arrows both occur. When this is the case, P(T)=P(D), where P(D) is described in Section 2. If clinicians think that a diagnostic test’s positive result is “necessary, but not sufficient” to confirm the presence of the disease, then that is equivalent to them accepting a diagnostic test that is actually inadequate at identifying the disease. The test either should be refined or replaced. We suggest it is imperative that clinicians continue their investigations—ultimately seeking a diagnostic test that consistently *does* identify the disease (Section 3.2).
(ii) Treat P(T) as a cumulative lifetime risk. Framing accurate diagnosis as a cumulative lifetime risk has implications for clinicians considering the usefulness of a diagnostic test result, as well as for developing long-term clinical studies (Section 3.3).

These two essential features make it more likely that unbiased clinical studies produce an estimator $\hat{P}\left(T\right)$ that is close to the estimator $\hat{P}\left(D\right)$ described in Section 2.2.

In order to be useful in diagnosis, all diagnostic tests must, within reasonable error bounds, give the same diagnostic information. At present, antibody tests, pregnancy tests, and blood tests for particular substances are examples of diagnostic tests for which high sensitivity and specificity determinations are standard. This standard should be applied to all tests (e.g., tissue biopsies) that are part of the diagnostic system. Even so, for some genetic diseases, not all subjects with the disease-causing genotype will appear to have the disease. This may be because of partial penetrance, but it should also be considered that incomplete diagnosis may be at fault or that people may tend to ignore their symptoms or ascribe them to other causes. Those persons should be more carefully followed up with additional investigations and perhaps different types of diagnostic tests.

Finally, we mention that when *G* and *T* are both necessary and sufficient for *D* (all blue and red arrows in Figure 7 occur), then

P(G)=P(D)=P(T),

and clinical studies should produce estimators for P(G) and P(T) that are close; that is, $\hat{P}\left(G\right)\approx \hat{P}\left(T\right)$. Because genetic tests are less likely to have errors than are diagnostic tests, a discrepancy between the estimators more than likely suggests that $\hat{P}\left(T\right)$ is not accurate, indicating that further investigation is warranted, rather than concluding simply that *G* is not sufficient.

### 5.3. Offspring-Group Aggregation (Section 4)

The general population can be partitioned into six offspring-groups denoted by $F_{i}$ (for i=1, 2, …, 6), and a specific offspring-group $F_{i}$ is determined by parental genotypes (Figure 6). We provide a theoretical framework for describing a genetic disease’s *offspring-group aggregation* (i.e., disease aggregation among the six offspring-groups).

We discuss the theoretical and computational deficiencies of the sibling recurrence-risk ratio, whose definition often is misunderstood and subject to differing and inconsistent interpretations. This ratio typically is used as an indicator of familial aggregation even though it ignores the six offspring-groups (Section 4.1).

We propose replacing familial aggregation with offspring-group aggregation, as well as an alternative measure that does not experience the deficiencies and precisely describes the frequency distribution of offspring-group aggregation among the six offspring-groups (Section 4.2). In summary, our proposed measure is the *offspring-group recurrence-risk ratio* (denoted by $\mu_{i}$), which is defined in Equation (9). It measures the likelihood a person from offspring-group $F_{i}$ has the disease, relative to a person from the general population. The frequency distribution of offspring-group aggregation is described by the equation

$$\sum_{i=1}^{6}P\left(F_{i}\right)\mu_{i}=1,$$

where $P\left(F_{i}\right)\mu_{i}$ is the offspring-group proportion of those with the disease who are in offspring-group $F_{i}$.

Finally, we note that $\mu_{i}$ and $P(F_{i})$ depend on understanding the disease-causing genotypes and the presence of the disease (Section 2), as well as accurate diagnosis of the disease (Section 3). Thus, our theoretical framework for offspring-group aggregation fundamentally relies on an understanding of the relationships between *G*, *D*, and *T*, as communicated by the green arrows in Figure 7.

**Offspring-group aggregation as a clinical tool**. We conclude with a scenario illustrating how a clinician may use the theoretical framework for offspring-group aggregation as a clinical tool. Consider a disease caused by a dominant allele with p=0.2, r=1, and P(D|CC)=1. Then, P(D)=0.36 (Equation (3)). Suppose a person visits a clinician wanting to know the likelihood they have the disease, given the person has a sibling known to have the disease. While the clinician may not know to which offspring-group the siblings belong, it is known they are not in offspring-group $F_{6}$. As illustrated in Table 3, the clinician predicts the person is either 1.39, 2.08, or 2.78 times as likely to have the disease, compared to members of the general population, which is 0.36. Using this information, the clinician predicts the likelihood that the person has the disease is approximately either 0.50, 0.75, or 1.00, respectively, and the person’s offspring-group determines which of the three values it is. However, even if the clinician does not know the person’s offspring-group, it is still possible to estimate the likelihood the person has the disease. Indeed, based on Table 4, the clinician notices that, of those people with the disease, $F_{5}$ has the highest percentage (in fact, higher than the sum of all other offspring-groups); thus, the clinician may choose to only use the $F_{5}$ information and predict that the likelihood the person has the disease is about (1.39)×(0.36)=0.50. Alternatively, the clinician may choose to use a weighted average, incorporating all the information in Table 3 and Table 4,

0.57(0.50)+0.21(0.75)+0.22(1.00)=0.66

as a prediction of the likelihood the person has the disease. Whichever value the clinician chooses (0.50 or 0.66), the clinician concludes the person is at a higher risk than a member of the general population (0.36). This information can be used to frame a discussion between the clinician and the patient regarding the next steps to pursue (e.g., whether to test the person for the disease-causing genotypes or administer accurate diagnostic tests).

We recommend that researchers and clinicians consider using the theoretical framework for offspring-group aggregation discussed in Section 4 and summarized in Section 5.3.

To place our analysis in the context of the current state of research, it is still epidemiologically valid to say that if one person in a hereditary family has a genetic disease, other family members are at risk, should be carefully evaluated, and appropriate precautions should be taken. Though other hereditary family members often are at higher risk than are members of the population as a whole, this does not mean $K_{s}>K$ in the general population. We suggest this mistaken idea be replaced by an approach that carefully uses diagnostic tools to accurately evaluate *K*, as well as describe genetic disease aggregation in terms of the offspring-groups $F_{i}$ and the offspring-group recurrence-risk ratio $\mu_{i}$.

## Figures and Tables

**Table 1:** The partition of a group of individuals by *D* and *T*.

	*T*	$T^{\prime }$
*D*	$n_{11}$	$n_{12}$
$D^{\prime }$	$n_{21}$	$n_{22}$

**Table 2:** All possible parental genotypes and frequencies of their offspring.

	CC	Cc	cC	cc
CC	$p^{2}\times p^{2}$	$p^{2}\times pq$	$p^{2}\times qp$	$p^{2}\times q^{2}$
Cc	$pq\times p^{2}$	pq×pq	pq×qp	$pq\times q^{2}$
cC	$qp\times p^{2}$	qp×pq	qp×qp	$qp\times q^{2}$
cc	$q^{2}\times p^{2}$	$q^{2}\times pq$	$q^{2}\times qp$	$q^{2}\times q^{2}$

**Table 3:** Illustrative examples of the offspring-group recurrence-risk ratio.

	p=0.2, r=1	p=0.2, r=0.5	p=0.02, r=1
$\mu_{1}$	2.78	5.00	25.25
$\mu_{2}$	2.78	3.75	25.25
$\mu_{3}$	2.78	2.50	25.25
$\mu_{4}$	2.08	2.50	18.94
$\mu_{5}$	1.39	1.25	12.63
$\mu_{6}$	0	0	0

**Table 4:** Offspring-group proportions when p=0.2 and r=1.

$P\left(F_{1}\right)\mu_{1}$	0.004
$P\left(F_{2}\right)\mu_{2}$	0.071
$P\left(F_{3}\right)\mu_{3}$	0.142
$P\left(F_{4}\right)\mu_{4}$	0.213
$P\left(F_{5}\right)\mu_{5}$	0.569
$P\left(F_{6}\right)\mu_{6}$	0

## Data Availability

Not applicable.

## Appendix A. Derivation of Equation (2)

Consider the partition of the population in terms of the genotypes CC, Cc, cC, and cc. Now,

D=(D∩CC)∪(D∩Cc)∪(D∩cC)∪(D∩cc),

and because the genotypes are mutually exclusive (the intersection of any two genotypes is the empty set *∅*),

P(D)=P(D∩CC)+P(D∩Cc)+P(D∩cC)+P(D∩cc).

Because P(D∩Cc)=P(D∩cC), we obtain

P(D)=P(D∩CC)+2P(D∩Cc)+P(D∩cc).

By the definition of the probability of an intersection,

P(D)=P(D|CC)P(CC)+2P(D|Cc)P(Cc)+P(D|cc)P(cc),

which can be written in the form shown in Equation (2).

## Appendix B. Necessary and Sufficient as Conditional Probabilities

We now develop equivalent conditional probability formulations for the concepts of “necessary” and “sufficient”. The formulations apply to any two events, but we will frame the discussion in terms of *G* and *D* (Section 2.3).

Observe that P(G|D)=1 is equivalent to saying that “*G* is *necessary* for *D*”. Indeed:

$$\begin{array}{cc} P(G|D)=1 & \Leftrightarrow P(D\cap G)=P(D)\\ \; & \Leftrightarrow P(D\cap G^{\prime })=0\;(\mathrm{because}\;P\left(D\right)=P\left(D\cap G\right)+P\left(D\cap G^{\prime }\right))\\ \; & \Leftrightarrow D\cap G^{\prime }=\emptyset \;(\emptyset \;\mathrm{denotes}\;\mathrm{the}\;\mathrm{empty}\;\mathrm{set})\\ \; & \Leftrightarrow D=D\cap G\;(\mathrm{because}\;D=\left(D\cap G\right)\cup \left(D\cap G^{\prime }\right))\\ \; & \Leftrightarrow \mathrm{the}\;\mathrm{occurrence}\;\mathrm{of}\;D\;\mathrm{implies}\;\mathrm{the}\;\mathrm{occurrence}\;\mathrm{of}\;G\\ \; & \Leftrightarrow G\;\mathrm{is}\;\mathrm{necessary}\;\mathrm{for}\;D. \end{array}$$

Furthermore, observe that P(D|G)=1 is equivalent to saying that “*G* is *sufficient* for *D*.” Indeed:

$$\begin{array}{cc} P(D|G)=1 & \Leftrightarrow P(G\cap D)=P(G)\\ \; & \Leftrightarrow P(G\cap D^{\prime })=0\;(\mathrm{because}\;P\left(G\right)=P\left(G\cap D\right)+P\left(G\cap D^{\prime }\right))\\ \; & \Leftrightarrow G\cap D^{\prime }=\emptyset ;\\ \; & \Leftrightarrow G=G\cap D\;(\mathrm{because}\;G=\left(G\cap D\right)\cup \left(G\cap D^{\prime }\right))\\ \; & \Leftrightarrow \mathrm{the}\;\mathrm{occurrence}\;\mathrm{of}\;G\;\mathrm{implies}\;\mathrm{the}\;\mathrm{occurrence}\;\mathrm{of}\;D\\ \; & \Leftrightarrow G\;\mathrm{is}\;\mathrm{sufficient}\;\mathrm{for}\;D. \end{array}$$

## Appendix C. Derivation of Equation (4)

Because $T=\left(T\cap D\right)\cup \left(T\cap D^{\prime }\right)$ and because *D* and $D^{\prime }$ are mutually exclusive,

$$\begin{array}{cc} P(T) & =P\left(T\cap D\right)+P\left(T\cap D^{\prime }\right)\\ \; & =P\left(T|D\right)P\left(D\right)+P\left(T|D^{\prime }\right)P\left(D^{\prime }\right)\\ \; & =P\left(D\right)+P\left(T|D^{\prime }\right)\left(1-P\left(D\right)\right)\;(\mathrm{because}\;P(T|D)=1) \end{array}$$

which implies,

$$\begin{array}{cc} 1-1P(T) & =\left(1-P\left(T|D^{\prime }\right)\right)P\left(D\right)+P\left(T|D^{\prime }\right)\;. \end{array}$$

Solving for P(D) yields

$$P\left(D\right)=\frac{P\left(T\right)-P\left(T|D^{\prime }\right)}{1-P(T|D^{\prime })}$$

To simplify the notation in the following derivation, we let ω=P(T) and $\alpha =P(T|D^{\prime })$. Then, we can write

$$\begin{array}{cc} P(D) & =\frac{\omega -\alpha }{1-\alpha }\\ \; & =\frac{\omega (1-\alpha )-\alpha (1-\omega )}{1-\alpha } \end{array}$$

which implies that

(A1) $$P\left(D\right)=\omega -\left(1-\omega \right)\frac{\alpha }{1-\alpha }\;\cdot$$

Let f(α)=α/(1−α) for 0≤α<1. The derivative of *f* is

$$f^{\prime }\left(\alpha \right)={\left(1-\alpha \right)}^{-2}>0\;,$$

which implies that f(α) is an increasing function on the interval 0≤α<1. Thus, for $\alpha_{0}$ with $0<\alpha_{0}<1$,

$$\begin{array}{cc} 0\leq \alpha \leq \alpha_{0} & \Rightarrow f\left(0\right)\leq f\left(\alpha \right)\leq f\left(\alpha_{0}\right)\\ \; & \Rightarrow 0\leq \frac{\alpha }{1-\alpha }\leq \frac{\alpha_{0}}{1-\alpha_{0}}. \end{array}$$

Therefore, using Equation (A1), we obtain the following lower and upper bounds for P(D):

$$\omega -\left(1-\omega \right)\frac{\alpha_{0}}{1-\alpha_{0}}\leq P\left(D\right)\leq \omega .$$

Substituting ω=P(T) yields Equation (4).
